# Template-independent synthesis and 3′-end labelling of 2′-modified oligonucleotides with terminal deoxynucleotidyl transferases

**DOI:** 10.1093/nar/gkae691

**Published:** 2024-08-16

**Authors:** Leping Sun, Yuming Xiang, Yuhui Du, Yangming Wang, Jiezhao Ma, Yaxin Wang, Xueting Wang, Guangyuan Wang, Tingjian Chen

**Affiliations:** MOE International Joint Research Laboratory on Synthetic Biology and Medicines, School of Biology and Biological Engineering, South China University of Technology, 510006 Guangzhou, China; MOE International Joint Research Laboratory on Synthetic Biology and Medicines, School of Biology and Biological Engineering, South China University of Technology, 510006 Guangzhou, China; MOE International Joint Research Laboratory on Synthetic Biology and Medicines, School of Biology and Biological Engineering, South China University of Technology, 510006 Guangzhou, China; MOE International Joint Research Laboratory on Synthetic Biology and Medicines, School of Biology and Biological Engineering, South China University of Technology, 510006 Guangzhou, China; MOE International Joint Research Laboratory on Synthetic Biology and Medicines, School of Biology and Biological Engineering, South China University of Technology, 510006 Guangzhou, China; MOE International Joint Research Laboratory on Synthetic Biology and Medicines, School of Biology and Biological Engineering, South China University of Technology, 510006 Guangzhou, China; MOE International Joint Research Laboratory on Synthetic Biology and Medicines, School of Biology and Biological Engineering, South China University of Technology, 510006 Guangzhou, China; MOE International Joint Research Laboratory on Synthetic Biology and Medicines, School of Biology and Biological Engineering, South China University of Technology, 510006 Guangzhou, China; MOE International Joint Research Laboratory on Synthetic Biology and Medicines, School of Biology and Biological Engineering, South China University of Technology, 510006 Guangzhou, China

## Abstract

Xenobiotic nucleic acids (XNAs) are artificial genetic polymers with altered structural moieties and useful features, such as enhanced biological and chemical stability. Enzymatic synthesis and efficient labelling of XNAs are crucial for their broader application. Terminal deoxynucleotidyl transferases (TdTs) have been exploited for the *de novo* synthesis and labelling of DNA and demonstrated the capability of recognizing various substrates. However, the activities of TdTs for the synthesis and labelling of commonly used XNAs with 2′ modifications have not been systematically explored. In this work, we explored and demonstrated the varied activities of three TdTs (bovine TdT, MTdT-evo and murine TdT) for the template-independent incorporation of 2′-methoxy NTPs, 2′-fluoro NTPs and 2′-fluoroarabino NTPs into the 3′ ends of single- and double-stranded DNAs and the extension of 2′-modified XNAs with (d)NTPs containing a natural or unnatural nucleobase. Taking advantages of these activities, we established a strategy for protecting single-stranded DNAs from exonuclease I degradation by TdT-synthesized 2′-modified XNA tails and methods for 3′-end labelling of 2′-modified XNAs by TdT-mediated synthesis of G-quadruplex-containing tails or incorporation of nucleotides with a functionalized nucleobase. A DNA-2′-fluoroarabino nucleic acid (FANA) chimeric hydrogel was also successfully constructed based on the extraordinary activity of MTdT-evo for template-independent FANA synthesis.

## Graphical Abstract

**Graphical Abstract:**
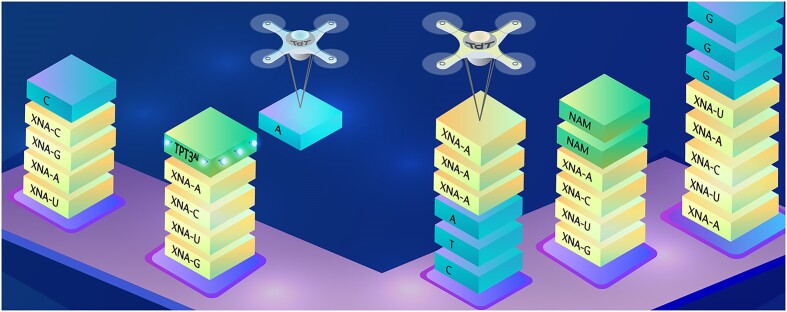

## Introduction

Xenobiotic nucleic acids (XNAs) are a series of artificial genetic polymers that have been developed by introducing modifications or totally unnatural moieties into the structural scaffolds of DNA and RNA (1–3). XNAs possess unique structures and sometimes very useful features, such as relatively high biological and chemical stability, which greatly expands the scope and application of genetic materials (4). For XNAs with altered sugar backbones, among the most common modifications are the substitutions at the 2′ position of ribose or deoxyribose, including 2′-methoxy (2′-OMe), 2′-fluoro (2′-F), 2′-amino (2′-Am), 2′-azido (2′-Az) and 2′-fluoroarabino (2′-F-ara or fa), which usually lead to significant changes in many properties of DNA and RNA (5–8). 2′-F and 2′-OMe modifications greatly increase the melting temperatures, duplex stabilities and nuclease resistance of nucleic acids, and thus have been widely used in the generation of stable aptamers or catalysts (6,9,10). 2′-F-ara modification of DNA leads to the production of 2′-fluoroarabinonucleic acid (FANA), which has higher resistance to acidic hydrolysis and nuclease degradation and the capability of recruiting RNase H when bound to RNA (11,12). It was also found that 2′-F-ara modification significantly affects the *T*_m_ values of DNA secondary structures (13), and FANA–DNA chimeras have good serum stability (14). Due to these properties, FANA has been extensively used to produce aptamers, catalysts and gene silencing agents with improved performances (10,15–19). In addition, FANA has a great potential in the creation of novel biosensors and nanomaterials (4,11,20,21).

Easy availability of XNA oligonucleotides is a prerequisite for their broad application, while traditional solid-phase synthesis of oligonucleotides has obvious disadvantages, such as limited product length and purity, the need for unstable phosphoramidites, harsh and environmentally unfriendly reaction conditions and high cost for the synthesis of modified oligonucleotides. Enzymatic synthesis provides a fascinating alternative for the preparation of XNA oligonucleotides, which can be carried out with relatively stable nucleoside triphosphates (NTPs) under milder and greener conditions and produce products with greater length and higher purity (22). One way to enzymatically produce XNA oligonucleotides is using a template-dependent polymerase to synthesize the XNA oligonucleotides from a DNA template. To realize this process with good efficiency, a great deal of effort has been made to obtain efficient XNA polymerases by engineering various DNA and RNA polymerases, including the Stoffel fragment of Taq DNA polymerase, Tgo DNA polymerase, KOD DNA polymerase, phi29 DNA polymerase and T7 RNA polymerase (23–29). Taking advantage of the engineered XNA polymerases, several strategies have also been developed for the mass production of XNA oligonucleotides (30–33). However, all these approaches synthesize XNA oligonucleotides in a template-dependent manner, and the synthesized XNA strand has to be purified away from the DNA template after synthesis, posing a limitation to the broad use of these approaches. Meanwhile, relatively less investigation has been carried out on the template-independent enzymatic synthesis of XNA oligonucleotides, which not only avoids the need for a DNA template, but may also have good compatibility with modern platforms for enzymatic *de novo* DNA synthesis (22).

Efficient labelling of nucleic acids is crucial for many of their applications, ranging from development of biosensors to production of nucleic acid–drug conjugates (34). 3′-End labelling is one of the most common and useful ways of labelling DNA and RNA, and can be done in a post-synthesis manner with proper enzymes (35–38). However, little effort has been made to develop methods for efficient 3′-end labelling of XNAs, which greatly limits the wide application of XNAs.

Terminal deoxynucleotidyl transferases (TdTs) belong to the X family of DNA polymerases (39) and can template-independently incorporate random deoxynucleotides into the 3′ end of single-stranded DNAs (ssDNAs). Due to this unique activity, TdTs have been extensively employed for the enzymatic *de novo* synthesis of DNA in recent years (40–45). Notably, a variety of novel biosensing tools and materials have also been developed based on the activity of TdTs (46–49). TdTs have demonstrated a broad substrate spectrum and can recognize a series of dNTP analogues, including biotinylated dNTPs (50), redox-active aminophenyl- or nitrophenyl-modified dNTPs (51), fluorescent benzo-expanded dNTPs (52), imidazole dNTP (dImTP) (53), carboxy-imidazole-modified dNTP (dIm^C^TP) (54), locked nucleic acid (LNA) NTPs (55) and bridged nucleic acid (BNA) NTPs (56). Taking advantage of some of these activities, enzymatic 3′-end labelling of DNA has been realized with TdTs (50,57–60). Moreover, we have previously reported the efficient template-independent incorporation of nucleotides with the unnatural nucleobase dTPT3 or dNaM into the 3′ end of ssDNAs via bovine TdT (61). The efficient incorporation of dTPT3 derivatives with different functional linkers into oligonucleotides by this TdT for orthogonal labelling of DNA and its subsequent applications has also been demonstrated. Despite all the explorations and applications of different TdT activities, the activities of different TdTs and their engineered mutants for the template-independent synthesis and 3′-end labelling of XNAs with various 2′ modifications have not been systematically investigated and compared.

In this work, we first explored the activities of three TdTs, bovine TdT (62), MTdT-evo (a variant of the polymerase domain of TdT from *Mus musculus* with enhanced thermostability, engineered by fusion to an N-terminal maltose-binding protein and a 10× His-tag and introduction of 15 mutations predicted by the FireProt algorithm) (63) and murine TdT (64), for the incorporation of three representative 2′-modified NTPs, namely 2′-OMe-NTPs, 2′-F-NTPs and 2′-F-ara-NTPs (faNTPs) into the 3′ ends of ssDNA and double-stranded DNA (dsDNA). The bivalent metal ions supplemented in the reaction solution and reaction time were then optimized for the MTdT-evo-mediated incorporation of these 2′-modified NTPs. The effect of the 3′-end nucleotide of the ssDNA primer on MTdT-evo-mediated incorporation of 2′-modified NTPs was also explored. Steady-state kinetic experiments were carried out to characterize and compare the activities of bovine TdT, MTdT-evo and murine TdT for the incorporation of different 2′-modified NTPs into the 3′ end of ssDNA. Based on MTdT-evo's activity towards 2′-modified NTPs, we then evaluated the exonuclease resistances of ssDNAs with 2′-modified nucleotides incorporated into their 3′ ends by MTdT-evo to establish a convenient method for the robust protection of ssDNA. We next explored the capacities of the three TdTs for the incorporation of (d)NTPs containing a natural or unnatural nucleobase into the 3′ ends of 2′-F-DNA, 2′-OMe-DNA and DNA with both 2′-OMe and 2′-F modifications (2′-OMe/F-DNA). Taking advantage of the efficient TdT incorporation of (d)NTPs into the 3′ end of 2′-F-DNA, we established a method for the 3′-end labelling of 2′-F-DNA by the MTdT-evo-mediated synthesis of a 3′ tail containing G-quadruplexes. To achieve convenient labelling or coupling of XNAs with unlimited functionalities, we also explored and demonstrated the activities of the three TdTs for the incorporation of (d)NTP derivatives harboring a functionalized natural or unnatural nucleobase into the 3′ ends of different XNAs. The time courses of MTdT-evo-mediated XNA extension with different functionalized (d)NTP derivatives were then investigated. In addition, employing MTdT-evo's activities towards faNTPs, a DNA–FANA chimeric hydrogel was constructed, broadening the application scope of the template-independent XNA synthesis by TdTs.

## Materials and methods

### Materials

Plasmids pET30a-bovine TdT, pET28a-MTdT-evo and pET28a-murine TdT, 5′-6-carboxyfluorescein (FAM)-labelled DNA primer FAM-18G, FAM-18A, FAM-18T and FAM-18C, DNA primers 18G-RC, X_1_, X_2_, X_3_ and X_4_, 2′-modified DNA primers F30 and MF25 (Supplementary Table S1) and 2× TBE-urea sample buffer were purchased from Sangon Biotech Co., Ltd (Shanghai, China). Kanamycin, thioflavin T (ThT), cobalt chloride (CoCl_2_), manganese chloride (MnCl_2_), magnesium acetate [Mg(OAc)_2_], cupric sulfate (CuSO_4_) and ascorbic acid were purchased from Solarbio Science & Technology Co., Ltd (Beijing, China). Isopropyl-β-d-thiogalactoside (IPTG) and the Magen HiPure gel pure DNA micro kit were purchased from newProbe Biotechnology Co., Ltd (Beijing, China). The BCA protein assay kit was purchased from Beyotime Biotechnology Co., Ltd (Shanghai, China). 2′-OMe-NTPs, 2′-F-NTPs, faNTPs, 5-propargylamino-dCTP (dC^Am^TP) and 5-ethynyl-dUTP (EdUTP) were purchased from Huaren Science & Technology Co., Ltd (Wuhu, China). dNaMTP, NaMTP, dTPT3TP, TPT3TP, dTPT3^Am^TP, dTPT3^Al^TP and TPT3^Al^TP were purchased from WuXi AppTec (Wuxi, China). dTPT3^Bio^TP was prepared in the lab. Zymo ssDNA/RNA Clean & Concentrator™ kit was purchased from Zymo Research (Irvine, CA, USA). Exo I was purchased from New England Biolabs (Ipswich, MA, USA). Cyber gold was purchased from Biolite Biotech Co., Ltd (Xi’an, China). The 1 kb DNA ladder was purchased from Yeasen Biotechnology Co., Ltd (Shanghai, China). The low molecular weight ladder was purchased from Dongsheng Biotech Co., Ltd (Guangzhou, China). Tris(3-hydroxypropyltriazolylmethyl)amine (THPTA) was purchased from Xi’an Confluore Biological Technology Co., Ltd (Xi’an, China). AF488-azide was purchased from Vector Laboratories Inc. (Newark, CA, USA). Plasmids pET30a-bovine TdT, pET28a-MTdT-evo and pET28a-murine TdT were transformed into *Escherichia coli* BL21(DE3) cells for the expression of TdTs (Supplementary Note S1 and S2).

### Extension of ssDNA primers with 2′-modified NTPs by TdTs

The primer extension was carried out by mixing 100 nM primer FAM-18G, 10 μM 2′-modified NTP, 1 mM CoCl_2_ and 0.1 μM bovine TdT, MTdT-evo or murine TdT in 1× TdT buffer (100 mM KCl, 30 mM Tris-acetate, 0.05% Triton X-100, pH 7.5). The mixture was incubated at 37°C for 30 min. For the optimization of metal ions for the primer extension, 1 mM CoCl_2_ in the reaction mixture was replaced with 1 mM MnCl_2_, 10 mM Mg(OAc)_2_ or one of different combinations of 1 mM CoCl_2_, 1 mM MnCl_2_ and 10 mM Mg(OAc)_2_, and 10 μM of one of the 2′-modified CTPs was used as the NTP substrate. For the optimization of the reaction time, the optimized reaction mixture was incubated at 37°C for different times. For the exploration of the effect of the 3′-end nucleotide of the ssDNA primer on MTdT-evo-mediated incorporation of 2′-modified NTPs, 100 nM primer FAM-18A, FAM-18T, FAM-18C or FAM-18G, 10 μM 2′-modified NTP, 1 mM CoCl_2_ and 0.1 μM MTdT-evo were mixed in 1× TdT buffer, and incubated at 37°C for 30 min. All the reactions were quenched by the addition of an equal volume of 2× TBE-urea sample buffer and incubation at 95°C for 10 min. The products were analysed with 20% denaturing polyacrylamide gel electrophoresis (PAGE) gels containing 8 M urea.

### Steady-state kinetic experiments for the incorporation of 2′-modified NTPs by different TdTs

The steady-state kinetic experiments were performed as described previously with minor modifications (61). A 2× reaction solution was prepared by mixing 200 nM primer FAM-18G, 0–128 μM 2′-modified NTP and 20 mM Mg(OAc)_2_ in 2× TdT buffer. A 5 μl aliquot of pre-warmed 2× reaction solution was mixed with 5 μl of bovine TdT, MTdT-evo or murine TdT of varying concentration, and incubated at 37°C for a certain time. The reaction was then quenched by the addition of 10 μl of 2× TBE-urea sample buffer and incubation at 95°C for 10 min. The products were analysed with 20% denaturing PAGE gels containing 8 M urea. All gel bands were quantified using Quantity One analysis software (Bio-Rad). *K*_m_ and *k*_cat_ values were calculated by fitting the data to the Michaelis–Menten equation with GraphPad Prism 8 software.

### Exo I resistance assay of ssDNAs 3′-end protected with 2′-modified nucleotides incorporated by MTdT-evo

To prepare an ssDNA 3′-end protected with 2′-modified nucleotides, 100 nM primer FAM-18G was mixed with 100 μM 2′-modified NTP, 1 mM CoCl_2_ and 1 μM MTdT-evo in 1× TdT buffer, and incubated at 37°C for 60 min. The extension products were purified with the Zymo ssDNA/RNA Clean & Concentrator™ kit according to the manufacturer's guidance. The purified products were mixed with 2 U/μl Exo I in 1× Exo I buffer and incubated at 37°C for 15 min. The digestion products were then mixed with an equal volume of 2× TBE-urea sample buffer, incubated at 95°C for 10 min and analysed with 20% denaturing PAGE gels containing 8 M urea.

### Extension of 3′ ends of dsDNAs with 2′-modified NTPs by TdTs

To produce the dsDNA substrate, primer FAM-18G was annealed with its reverse complementary oligonucleotide 18G-RC by mixing them in the ratio of 1:1.2 in 2× TdT buffer, incubating the mixture at 95°C for 10 min and then slowly cooling down to room temperature. dsDNA at 100 nM was mixed with 100 μM 2′-modified NTPs, 1 mM CoCl_2_ and 1 μM bovine TdT, MTdT-evo or murine TdT in 1× TdT buffer and incubated at 37°C for 60 min to extend the 3′ ends of the dsDNA. The extension products were mixed with an equal volume of 2× TBE-urea sample buffer, incubated at 95°C for 10 min and analysed with 20% denaturing PAGE gels containing 8 M urea.

### Extension of XNA primers with nucleoside triphosphates containing a natural or unnatural nucleobase by TdTs

A 100 nM concentration of 2′-F-DNA primer F30 or 2′-OMe/F-DNA primer MF25 (Supplementary Table S1) was mixed with 10 μM (d)NTP (dATP, dTTP, dCTP, dGTP, dNaMTP, dTPT3TP, ATP, UTP, CTP, GTP, NaMTP or TPT3TP), 1 mM CoCl_2_ and 0.1 μM bovine TdT, MTdT-evo or murine TdT in 1× TdT buffer, and incubated at 37°C for 30 min. The extension products were mixed with an equal volume of 2× TBE-urea sample buffer, incubated at 95°C for 10 min and analysed with 20% denaturing PAGE gels containing 8 M urea.

### 3′-End labelling of 2′-F-DNA by MTdT-evo-mediated synthesis of G-quadruplex-containing oligonucleotide tails

A 200 nM concentration of 2′-F-DNA was mixed with 100 μM of a single dNTP or mixed dNTPs (i.e. dGTP only, 60% dGTP + 40% dATP, 60% dGTP + 40% dTTP, 50% dGTP + 40% dATP + 10% dTTP and 50% dATP + 50% dTTP), 1 mM CoCl_2_ and 1 μM MTdT-evo in 1× TdT buffer and incubated at 37°C for 30 min. The products were purified by the Zymo ssDNA/RNA Clean & Concentrator™ kit, mixed with 100 μM ThT in 1× Tris–HCl buffer (50 mM Tris–HCl, 50 mM KCl, pH 7.2) and incubated at room temperature for 20 min. The fluorescence emission spectra of the resultant solutions were collected over the range of 465–695 nm with intervals of 10 nm and excitation at 425 nm (46).

### 3′-End labelling of XNAs by TdT-mediated incorporation of nucleotides containing a functionalized nucleobase

A concentration of 100 nM 2′-F-DNA primer F30 or 2′-OMe/F-DNA primer MF25 was mixed with 10 μM (d)NTP (dC^Am^TP, EdUTP, dTPT3^Al^TP, dTPT3^Am^TP, dTPT3^Bio^TP or TPT3^Al^TP), 1 mM CoCl_2_ and 0.1 μM bovine TdT, MTdT-evo or murine TdT in 1× TdT buffer and incubated at 37°C for 30 min. The extension products were mixed with an equal volume of 2× TBE-urea sample buffer, incubated at 95°C for 10 min and analysed with 20% denaturing PAGE gels containing 8 M urea.

For the investigation of the time courses of MTdT-evo-mediated XNA extension with different functionalized (d)NTP derivatives, 100 nM primer F30 or MF25 was mixed with 10 μM dC^Am^TP, EdUTP, dTPT3^Al^TP, dTPT3^Am^TP, dTPT3^Bio^TP or TPT3^Al^TP, 1 mM CoCl_2_ and 0.1 μM MTdT-evo in 1× TdT buffer and incubated at 37°C for 5–80 min. The extension products were mixed with an equal volume of 2× TBE-urea sample buffer, incubated at 95°C for 10 min and analysed with 20% denaturing PAGE gels containing 8 M urea.

For the 3′-end fluorescent labelling of 2′-F-DNA by MTdT-evo-mediated incorporation of TPT3^Al^TP and further modification of TPT3^Al^ by click reaction, 100 nM F30 was mixed with 10 μM TPT3^Al^TP, 1 mM CoCl_2_ and 0.1 μM MTdT-evo in 1× TdT buffer, and incubated at 37°C for 30 min. The extension product was purified with a Zymo ssDNA/RNA Clean & Concentrator™ kit, mixed with 100 μM CuSO_4_, 500 μM THPTA, 100 μM AF488-azide and 2.5 mM ascorbic acid, and incubated at 37°C for 30 min. After purification with a Zymo ssDNA/RNA Clean & Concentrator™ kit, the product of the click reaction was mixed with an equal volume of 2× TBE-urea sample buffer, incubated at 95°C for 10 min and analysed with a 20% denaturing PAGE gel containing 8 M urea.

### MTdT-evo-based preparation and characterization of the DNA–FANA chimeric hydrogel

The DNA–FANA chimeric hydrogel was prepared similarly to as described in a previous work (48). Briefly, four DNA primers, X_1_, X_2_, X_3_ and X_4_, were annealed by mixing 10 μM each of them in 5× TdT buffer, incubating the mixture at 95°C for 10 min and slowly cooling down to room temperature to produce the X-DNA that was used as the initiator for the polymerization of faNTPs mediated by MTdT-evo (65). Then 2 μM X-DNA was mixed with 5 mM faATP or faUTP, 10 mM Mg(OAc)_2_, 1 mM MnCl_2_ and 10 μM MTdT-evo in 1× TdT buffer. The mixture was incubated at 37°C for 12 h to extend the X-DNA. All the annealing and extension products were analysed with 1.5% agarose gels.

The X-DNAs extended respectively with faATP and faUTP were then prepared in large quantities and concentrated to 20 μM with a vacuum centrifugal concentrator by centrifugation at 4500 rpm and 45°C. X-DNA (20 μM) extended with faATP and X-DNA (20 μM) extended with faUTP were then mixed and annealed by incubating at 50°C for 10 min, slowly cooling down to room temperature and incubating at 4°C for 20 min to allow the formation of the DNA–FANA chimeric hydrogel. The hydrogel was stained with Cyber gold and imaged with a camera and a fluorescence microscope (Leica DM6B, Germany). The hydrogel was also imaged with a scanning electron microscope (SEM, ZEISS MERLIN Compact, Germany) after dehydration and gold sputtering.

## Results and discussion

### Varied activities of different TdTs for ssDNA extension with 2′-modified NTPs

**Figure 1.**
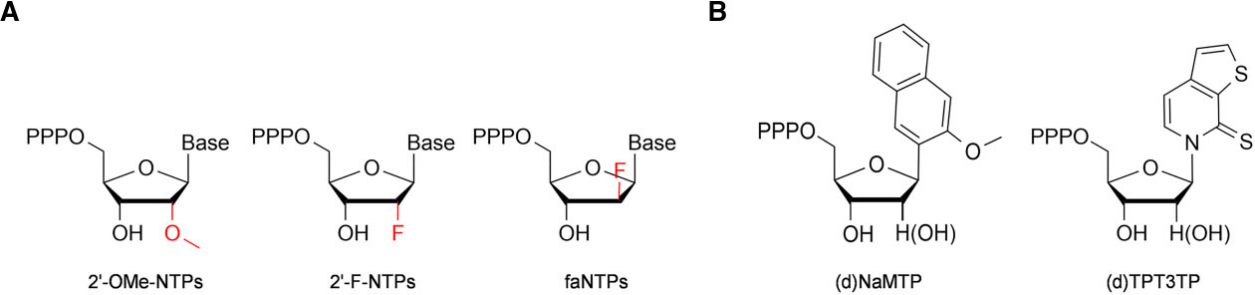
Chemical structures of 2′-modified NTPs (**A**) and (d)NTPs containing an unnatural nucleobase (**B**) used in this study.

**Figure 2.**
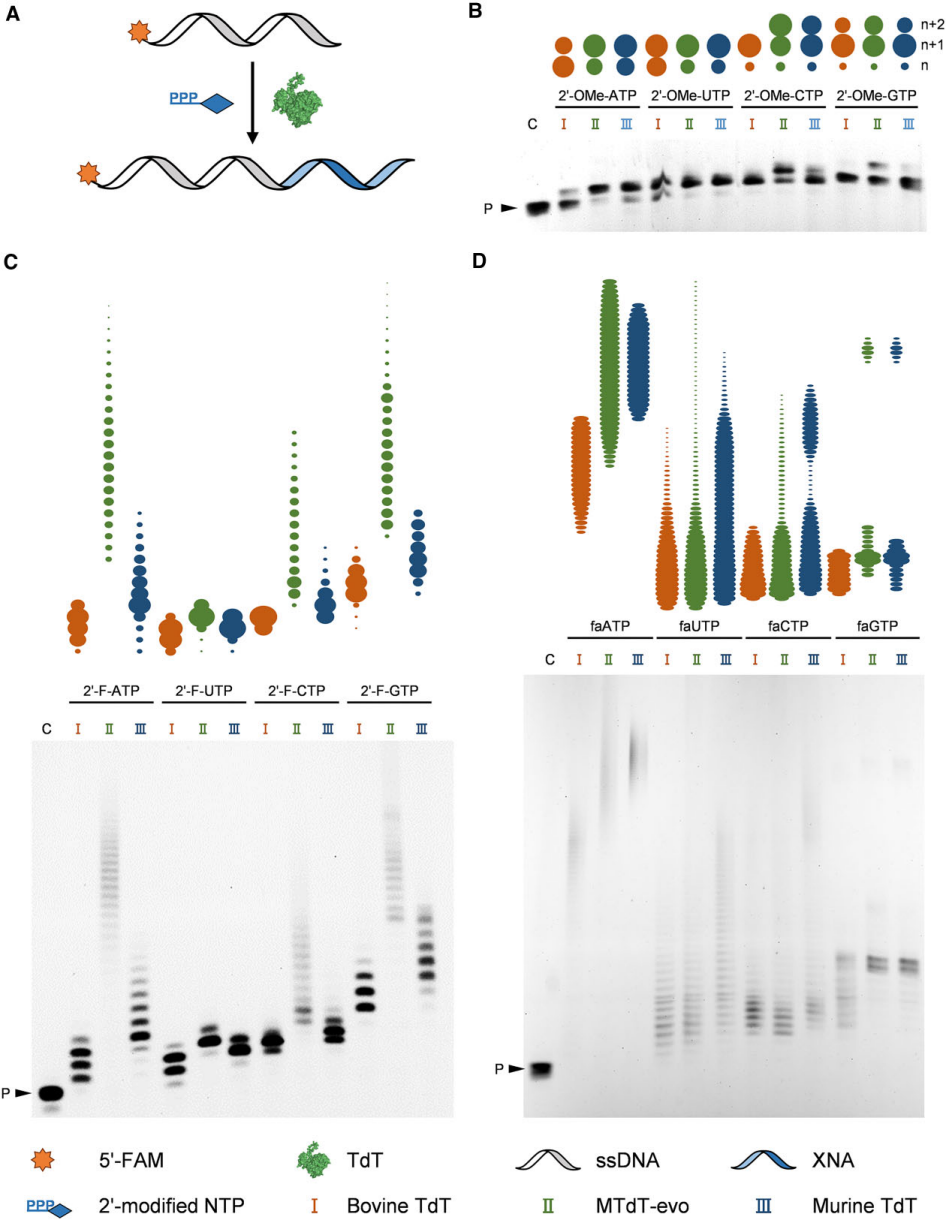
TdT-mediated extension of ssDNA with 2′-modified NTPs. (**A**) Scheme of the experiment. (**B**) Extension of ssDNA with different 2′-OMe-NTPs. (**C**) Extension of ssDNA with different 2′-F-NTPs. (**D**) Extension of ssDNA with different faNTPs. C, control (without the addition of TdT); P, primer FAM-18G; I, extension by bovine TdT; II, extension by MTdT-evo; III, extension by murine TdT. The product bands were quantified and presented as ovals above the gel images, and the area of each oval represents the intensity of the corresponding band.

TdTs have demonstrated the ability to incorporate a series of dNTPs and dNTP analogues into the 3′ end of ssDNA primers in the absence of a DNA template (50–55,61,66). To explore the activities of different TdTs towards 2′-modified NTPs, bovine TdT, MTdT-evo and murine TdT were employed to incorporate 2′-OMe-NTPs, 2′-F-NTPs and faNTPs (Figure 1A) to the 3′ end of an ssDNA primer (Figure 2A). The FAM-labelled ssDNA primer FAM-18G (Supplementary Table S1) was extended by mixing it with one of the 2′-modified NTPs, 1 mM CoCl_2_ and one of the three TdTs in 1× TdT buffer and incubating the mixture at 37°C for 30 min. As shown in Figure 2B–D, all three TdTs could incorporate one to many 2′-modified nucleotides into the 3′ end of the primer with all the tested 2′-modified NTPs. Clearly, the three TdTs have varying levels of activity towards different 2′-modified NTPs. In general, they all exhibited relatively high activity towards 2′-F-NTPs and faNTPs, especially 2′-F-GTP and faATP. However, all three TdTs exhibited lower levels of activity towards 2′-OMe-NTPs, which is likely to be due to a larger steric hindrance of the 2′-OMe group with the amino acid residues in the enzyme. Notably, under the tested conditions, for the incorporation of 2′-OMe-NTPs and 2′-F-NTPs, MTdT-evo demonstrated generally higher activity than the other two TdTs (Figure 2B, C), while for the incorporation of faNTPs, MTdT-evo and murine TdT demonstrated generally higher activity than bovine TdT (Figure 2D). In summary, among all TdTs, MTdT-evo exhibited the highest efficiency for the incorporation of most of the 2′-modified NTPs, and the TdT-mediated template-independent synthesis of FANA was found to be generally more efficient than those of 2′-F-DNA and 2′-OMe-DNA. Based on these results, MTdT-evo was selected for further optimization of the reaction conditions and demonstration of the applications of TdT-mediated XNA synthesis.

### Optimization of the reaction conditions for ssDNA extension with 2′-modified NTPs by MTdT-evo

Divalent metal ions are essential for the catalytic activities of TdTs, and the types and concentrations of these ions have proven to have significant effects on the efficiency of TdT-mediated nucleotide polymerization (67). Previously, we have investigated the effects of different metal ions, including Mg^2+^, Mn^2+^, Co^2+^ and Zn^2+^, and their combinations on the incorporation efficiencies of the unnatural dNTPs dNaMTP and dTPT3TP by bovine TdT (61). We found that Mg^2+^, Mn^2+^ and Co^2+^ had obvious effects on the incorporation efficiencies, and the combination of 10 mM Mg(OAc)_2_ and 1 mM MnCl_2_ was optimal. We herein investigated the effects of 1 mM CoCl_2_, 10 mM Mg(OAc)_2_, 1 mM MnCl_2_ and their combinations on the activities of MTdT-evo for extending the ssDNA primer FAM-18G with 2′-OMe-CTP, 2′-F-CTP and faCTP. As shown in Supplementary Figure S1A and B, 1 mM CoCl_2_ was optimal for the incorporation of both 2′-OMe-CTP and 2′-F-CTP by MTdT-evo, while faCTP could be efficiently incorporated by MTdT-evo with any of the metal ions or their combinations (Supplementary Figure S1C).

The time courses of MTdT-evo-mediated ssDNA extension with different 2′-modified NTPs were also investigated to provide references for reaction time determination in further experiments. Primer FAM-18G was extended with 10 μM of one of the 2′-modified NTPs by MTdT-evo in the presence of 1 mM CoCl_2_ for different reaction times, and the products were analysed with denaturing PAGE gels. As shown in Supplementary Figure S2A–C, generally, increasing numbers of 2′-modified nucleotides were incorporated into the 3′ end of the primer along with the increase of the reaction time in the tested range. In good agreement with the results of the preliminary activity test (Figure 2D), MTdT-evo exhibited good efficiency for the incorporation of faNTPs, especially faATP, and many nucleotides were incorporated after only 5 min (Supplementary Figure S2C). For the incorporation of faATP, the product length showed a more significant increase along with the increase of the reaction time. For the incorporation of 2′-F-NTPs, the incorporation of 2′-F-ATP and 2′-F-GTP was more efficient than that of 2′-F-UTP and 2′-F-CTP (Supplementary Figure S2B). The increase of the product length along with the increase of the reaction time was more significant for the incorporation of 2′-F-ATP and 2′-F-CTP. Interestingly, for the incorporation of faGTP and 2′-F-GTP, pauses in primer extension were observed, probably due to the formation of G-quadruplexes that impeded the incorporation of more nucleotides (13,63). For the incorporation of 2′-OMe-NTPs, within the tested reaction time, no more than three nucleotides had been incorporated for all four triphosphates (Supplementary Figure S2A). 2′-OMe-GTP and 2′-OMe-CTP were incorporated faster than 2′-OMe-UTP and 2′-OMe-ATP, and most of the primer (> 80%) was extended for at least one nucleotide with 2′-OMe-GTP and 2′-OMe-CTP after only 5 min.

Next, we explored the effect of the 3′-end nucleotide of the ssDNA primer on MTdT-evo-mediated incorporation of 2′-modified NTPs. Primers with different 3′-end nucleotides [FAM-18A, FAM-18T, FAM-18C and FAM-18G (Supplementary Table S1)] were extended with 10 μM of one of the 2′-modified NTPs by MTdT-evo in the presence of 1 mM CoCl_2_ at 37°C for 30 min, and the products were analysed with denaturing PAGE gels. As shown in Supplementary Figure S3A–C, under the tested conditions, the 3′-end nucleotide of the ssDNA primer demonstrated an obvious effect on the incorporation efficiencies of different 2′-modified NTPs by MTdT-evo. For 2′-OMe-NTPs, the incorporation efficiencies were lowest when primer FAM-18C was used. When primers FAM-18T and FAM18-G were used, the incorporation efficiencies were comparable and generally slightly higher than those when primer FAM-18A was used. For 2′-F-NTPs, the incorporation efficiencies were lowest when primer FAM-18A was used and comparable when primers FAM-18T and FAM-18G were used. When FAM-18C was used, the incorporation efficiencies of 2′-F-ATP and 2′-F-UTP were lower and the incorporation efficiencies of 2′-F-CTP and 2′-F-GTP were similar compared with those when primers FAM-18T and FAM-18G were used. For faNTPs, the incorporation efficiencies were lowest when primer FAM-18C was used and comparable when the other three primers were used.

### Steady-state kinetics for the incorporation of 2′-modified NTPs by different TdTs

To quantify the activities of different TdTs for the incorporation of 2′-modified NTPs, steady-state kinetic experiments were carried out. The fitted Michaelis–Menten curves are shown in Supplementary Figures S4–S15 and the calculated *K*_m_, *k*_cat_ and *k*_cat_/*K*_m_ values for the incorporation of 2′-modified NTPs and dNTPs mediated by different TdTs are summarized in Table 1. Generally, for the incorporation of each 2′-OMe-NTP, the catalytic efficiencies (reflected by the *k*_cat_/*K*_m_ values) of MTdT-evo and murine TdT are higher than those of bovine TdT. For the incorporation of each of the 2′-F-NTPs, the catalytic efficiency of murine TdT is higher than those of bovine TdT and MTdT-evo. For the incorporation of each of the faNTPs, the catalytic efficiencies of MTdT-evo and murine TdT are higher than those of bovine TdT. With only a few exceptions, for all the three TdTs, the order of the catalytic efficiencies for the incorporation of different 2′-modified NTPs with the same nucleobase is: faNTP > 2′-F-NTP > 2′-OMe-NTP. Surprisingly, although faNTPs are not natural substrates for the TdTs, for all three TdTs, the catalytic efficiency for the incorporation of each faNTP is comparable with or even higher than those for the incorporation of the corresponding dNTP. In addition, for murine TdT, the *k*_cat_/*K*_m_ values for the incorporation of 2′-F-NTPs were 3.07, 0.33, 0.30 and 1.06 times those for the incorporation of corresponding dNTPs, reflecting higher or close catalytic efficiencies for murine TdT-mediated incorporation of 2′-F-NTPs compared with those of dNTPs. It is noteworthy that for some TdTs and some 2′-modified NTPs, the catalytic efficiencies for the incorporation of a single 2′-modified nucleotide do not correlate well with the efficiencies of primer extension where multiple 2′-modified nucleotides are incorporated. For example, although the catalytic efficiencies for the MTdT-evo-mediated incorporation of 2′-OMe-CTP and 2′-F-CTP were fairly close, many nucleotides were incorporated for the primer extension with 2′-F-CTP, while only a few nucleotides were incorporated for that with 2′-OMe-CTP under the same reaction conditions (Figure 2; Supplementary Figure S2). This might be attributed to the following reasons. First, different 2′-modified nucleotides incorporated into the 3′ end of the primer might cause conformational changes to the 3′ end of the primer and impede the incorporation of more 2′-modified nucleotides to different extents. Second, the amino acid residues in the TdTs might have different levels of steric hindrance not only with the 2′ groups in different incoming 2′-modified NTPs but also with the 2′ groups in different 2′-modified nucleotides previously incorporated into the 3′ end of the primer.

**Table 1.** Steady-state kinetic constants for the incorporation of 2′-modified NTPs and dNTPs mediated by different TdTs

Nucleoside triphosphate	Bovine TdT			MTdT-evo			Murine TdT		
	*K*_m_ (μM)	*k*_cat_ (min^−1^)	*k*_cat_/*K*_m_ (mM^−1^ min^−1^)	*K*_m_ (μM)	*k*_cat_ (min^−1^)	*k*_cat_/*K*_m_ (mM^−1^ min^−1^)	*K*_m_ (μM)	*k*_cat_ (min^−1^)	*k*_cat_/*K*_m_ (mM^−1^ min^−1^)
2′-OMe-ATP	6.15 ± 1.85	0.12 ± 0.013	19.51	2.76 ± 0.62	0.08 ± 0.0043	28.99	3.58 ± 0.68	0.17 ± 0.014	47.49
2′-OMe-UTP	6.38 ± 0.96	0.0045 ± 0.00015	0.71	4.54 ± 1.17	0.05 ± 0.0038	11.01	2.82 ± 0.45	0.0075 ± 0.00086	2.66
2′-OMe-CTP	5.87 ± 0.76	0.024 ± 0.0035	4.09	6.21 ± 2.04	0.46 ± 0.0149	74.07	5.1 ± 1.53	0.067 ± 0.0052	13.14
2′-OMe-GTP	6.10 ± 1.50	0.041 ± 0.0013	6.72	1.41 ± 0.36	0.07 ± 0.0029	49.65	1.97 ± 0.14	0.13 ± 0.0052	65.99
2′-F-ATP	0.97 ± 0.27	0.56 ± 0.066	5.77 × 10^2^	6.08 ± 1.35	1.55 ± 0.16	2.55 × 10^2^	0.62 ± 0.48	1.38 ± 0.065	2.23 × 10^3^
2′-F-UTP	5.33 ± 1.58	0.16 ± 0.0067	30.02	5.24 ± 1.08	0.16 ± 0.02	30.53	0.95 ± 0.31	0.37 ± 0.034	3.89 × 10^2^
2′-F-CTP	8.42 ± 3.22	0.85 ± 0.043	1.01 × 10^2^	8.02 ± 1.34	0.44 ± 0.03	54.86	2.69 ± 0.88	2.02 ± 0.14	7.51 × 10^2^
2′-F-GTP	4.03 ± 0.52	1.09 ± 0.029	2.70 × 10^2^	12.82 ± 2.97	3.00 ± 0.25	2.34 × 10^2^	2.59 ± 0.36	5.35 ± 0.33	2.07 × 10^3^
faATP	2.23 ± 0.32	2.66 ± 0.43	1.19 × 10^3^	1.91 ± 0.17	15.30 ± 0.96	8.01 × 10^3^	3.98 ± 0.33	12.26 ± 0.17	3.08 × 10^3^
faUTP	2.63 ± 0.77	0.89 ± 0.039	3.38 × 10^2^	0.30 ± 0.07	7.29 ± 0.37	2.43 × 10^4^	2.36 ± 0.60	3.60 ± 0.26	1.53 × 10^3^
faCTP	2.83 ± 0.69	1.71 ± 0.19	6.04 × 10^2^	8.05 ± 0.61	7.46 ± 0.31	9.27 × 10^2^	1.66 ± 0.71	5.40 ± 0.40	3.25 × 10^3^
faGTP	2.91 ± 0.52	3.83 ± 0.25	1.32 × 10^3^	1.59 ± 0.27	16.08 ± 0.71	1.01 × 10^4^	2.75 ± 0.23	14.84 ± 0.42	5.40 × 10^3^
dATP	2.65 ± 0.76	0.31 ± 0.045	1.17 × 10^2^	1.33 ± 0.37	6.41 ± 0.34	4.82 × 10^3^	2.53 ± 0.30	1.84 ± 0.071	7.27 × 10^2^
dTTP	4.19 ± 0.91	0.45 ± 0.05	1.07 × 10^2^	2.85 ± 0.21	4.27 ± 0.18	1.50 × 10^3^	1.00 ± 0.33	1.19 ± 0.11	1.19 × 10^3^
dCTP	3.14 ± 0.46	1.20 ± 0.060	3.82 × 10^2^	1.95 ± 0.29	8.78 ± 0.35	4.50 × 10^3^	1.37 ± 0.18	3.48 ± 0.33	2.54 × 10^3^
dGTP	3.30 ± 1.93	0.96 ± 0.083	2.91 × 10^2^	2.39 ± 0.18	7.99 ± 0.41	3.34 × 10^3^	2.84 ± 0.84	5.55 ± 0.67	1.95 × 10^3^

### 3′-End protection of ssDNAs by MTdT-evo-mediated incorporation of 2′-modified nucleotides

**Figure 3.**
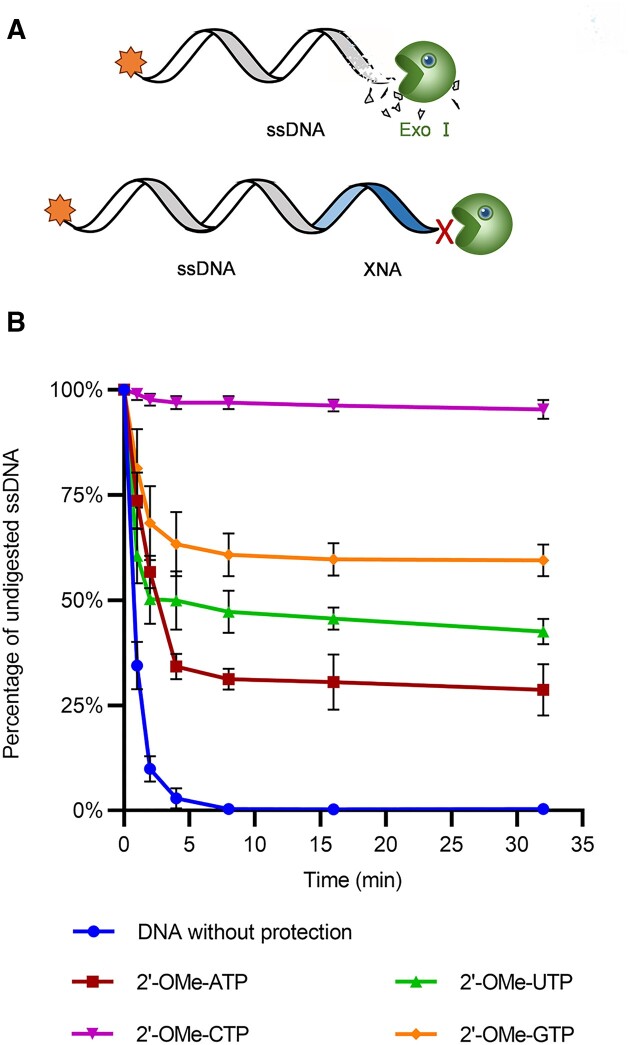
Protection of ssDNA against Exo I degradation by TdT-mediated 3′ tailing with 2′-OMe-NTPs. (**A**) Scheme of the experiment. (**B**) Time courses of Exo I degradation of ssDNAs with or without TdT-mediated 3′ tailing with one of the 2′-OMe-NTPs. The initial amount of DNA is defined as 100%. Error bars indicate standard deviations from the means (*n* = 3).

The introduction of 2′ modifications has been found to greatly increase the nuclease resistances of nucleic acids (7). Inspired by this fact and taking advantage of the capability of TdTs for incorporating 2′-modified NTPs, we attempted to establish a convenient method for 3′-end protection of ssDNAs from exonuclease degradation by tailing them with 2′-modified nucleotides using TdTs. To add the tails of different 2′-modified nucleotides, ssDNA primer FAM-18G was extended by mixing it with 0.1 mM of one of 2′-OMe-NTPs, 2′-F-NTPs or faNTPs, 1 mM CoCl_2_ and 1 μM MTdT-evo in 1× TdT buffer and incubating the mixture at 37°C for 60 min. To evaluate the protective effects of different 2′-modified nucleotide tails, the primer extension products were purified, mixed with 2 U/μl exonuclease I (Exo I, a 3′–5′ ssDNA exonuclease) and incubated at 37°C for 15 min. Denaturing PAGE analysis of the degradation products revealed that the Exo I resistances of ssDNAs tailed with different 2′-modified nucleotides were strikingly different (Supplementary Figure S16A–C). Generally, ssDNAs protected with 2′-OMe-modified nucleotides exhibited the highest resistances to Exo I. After digestion with Exo I for 32 min, 95, 61, 43 and 29% of the ssDNAs protected with 2′-OMe-C, 2′-OMe-G, 2′-OMe-U and 2′-OMe-A tails remained, respectively (Figure 3; Supplementary Figure S16D). In sharp contrast, the unprotected DNA was totally degraded after 8 min. Obviously, 2′-OMe-C and 2′-OMe-G tails protected the ssDNA better than 2′-OMe-U and 2′-OMe-A tails, probably due to more nucleotides incorporated by MTdT-evo when 2′-OMe-CTP and 2′-OMe-GTP were used for ssDNA tailing (Figure 2B). Remarkably, the addition of as few as two 2′-OMe-modified nucleotides could provide efficient protection to the ssDNA against Exo I degradation, indicating a convenient method for ssDNA protection by MTdT-evo-mediated incorporation of 2′-OMe-NTPs, which may also cause minimum disturbance to the ssDNA function. Notably, the addition of a long 2′-F-ara-A tail by MTdT-evo could also efficiently protect the ssDNA against Exo I degradation (Supplementary Figure S16C). Moreover, MTdT-evo-incorporated short stretches of 2′-F and 2′-F-ara-G nucleotides provided some protection to the ssDNA (Supplementary Figure S16B and Supplementary Figure S7C), which might be related to the formation of G-quadruplexes that could contribute to the enhanced stability and nuclease resistance of nucleic acids (68,69).

### Activities of different TdTs for 3′ tailing of dsDNA with 2′-modified NTPs

**Figure 4.**
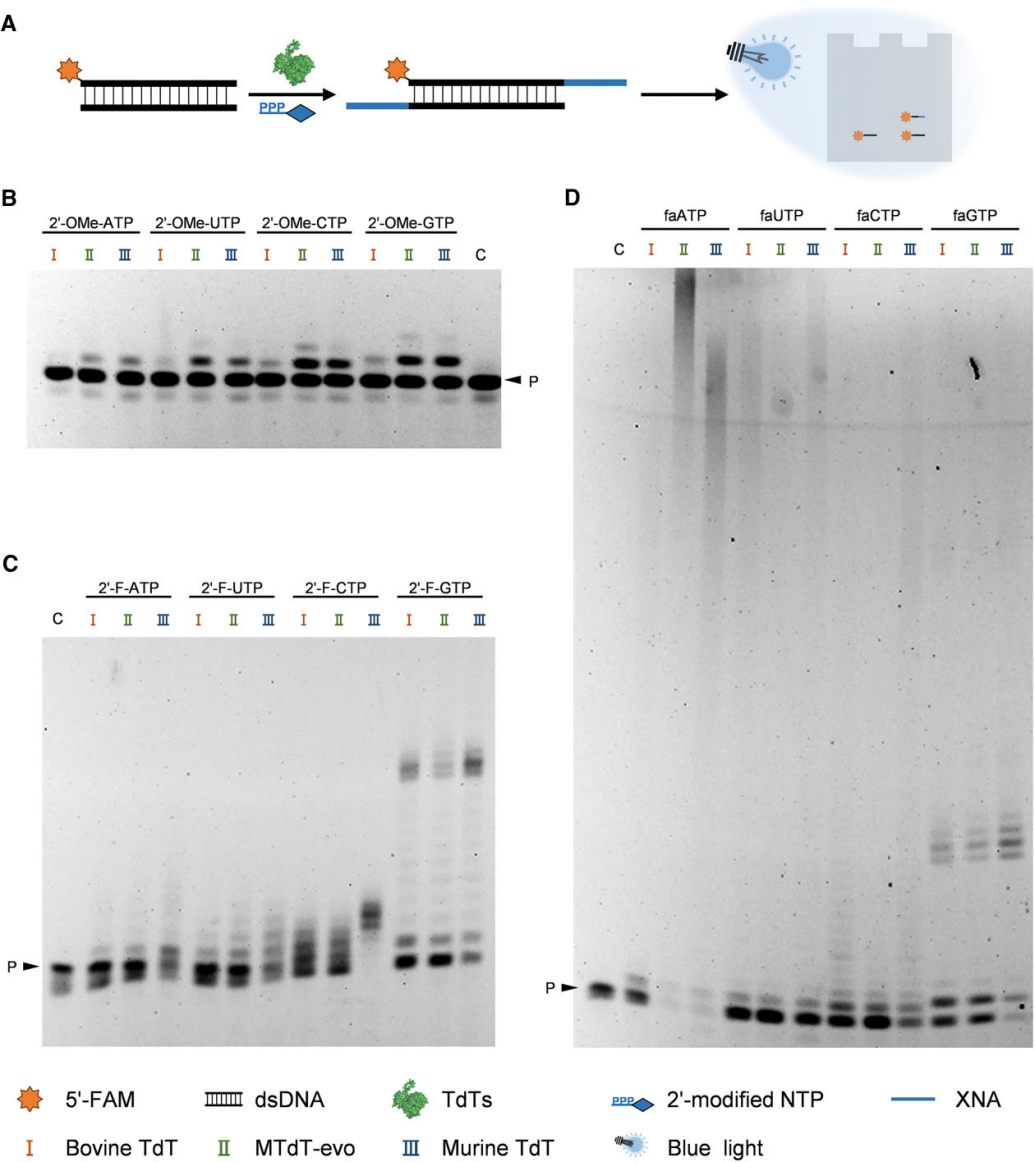
TdT-mediated 3′ tailing of dsDNA with 2′-modified NTPs. (**A**) Scheme of the experiment. (**B**) 3′ tailing of dsDNA with different 2′-OMe-NTPs. (**C**) 3′ tailing of dsDNA with different 2′-F-NTPs. (**D**) 3′ tailing of dsDNA with different faNTPs. C, control (without the addition of TdT); P, primer FAM-18G; I, 3′ tailing by bovine TdT; II, 3′ tailing by MTdT-evo; III, 3′ tailing by murine TdT.

TdTs have been found to be capable of incorporating deoxynucleotides into the 3′ ends of not only ssDNAs, but also dsDNAs (70). To test if TdTs can also incorporate 2′-modified nucleotides into the 3′ ends of dsDNAs, we carried out dsDNA tailing experiments with different 2′-modified NTPs using the three TdTs described above. Primer FAM-18G was annealed with its reverse complementary oligonucleotide 18G-RC (Supplementary Table S1) to produce the dsDNA substrate. The 3′ ends of the dsDNA substrate was then extended with different 2′-modified NTPs in the presence of 1 mM CoCl_2_ using bovine TdT, MTdT-evo and murine TdT, respectively (Figure 4A), and the extension products were analysed with denaturing PAGE gels. As shown in Figure 4B–D, most of the 2′-modified NTPs could be successfully incorporated into the 3′ ends of the dsDNA substrate by all three TdTs. Only for the incorporation of 2′-OMe-ATP with bovine TdT was no obvious extension product observed. Among all three TdTs, MTdT-evo exhibited the highest activity for the dsDNA extension with 2′-OMe-NTPs (Figure 4B), while murine TdT demonstrated the highest activity for the dsDNA extension with 2′-F-NTPs (Figure 4C). For the dsDNA extension with faNTPs, the types of the nucleobase had a larger effect on the relative activities of different TdTs (Figure 4D). MTdT-evo and murine TdT exhibited higher activities towards faATP than bovine TdT. All three TdTs exhibited similar activities towards faUTP. Murine TdT exhibited the highest activity towards faCTP and faGTP. Although MTdT-evo could efficiently incorporate many 2′-F-ara-C nucleotides into the 3′ end of an ssDNA primer, it could only incorporate a few 2′-F-ara-C nucleotides into the 3′ ends of the dsDNA substrate. Similarly, bovine TdT exhibited significant activity for the extension of ssDNA with faATP, but much less activity for the extension of dsDNA with faATP. Encouragingly, murine TdT could efficiently extend dsDNA with most of the faNTPs.

We also tested if TdT’s activity for dsDNA extension with 2′-modified NTPs could be improved by optimizing the metal ions supplemented into the reaction solution. MTdT-evo-mediated dsDNA extension with 2′-modified CTPs was chosen as the model for optimization. A 100 nM concentration of dsDNA substrate was mixed with 100 μM of one of 2′-modified CTPs, 1 mM CoCl_2_, 1 mM MnCl_2_, 10 mM Mg(OAc)_2_ or one of their combinations, and 1 μM MTdT-evo in 1× TdT buffer and incubated at 37°C for 60 min for dsDNA extension. The extension products were then analysed by denaturing PAGE. As shown in Supplementary Figure S17A–C, 1 mM CoCl_2_ was optimal for the MTdT-evo-mediated dsDNA extension with 2′-OMe-CTP, 1 mM MnCl_2_ was optimal for the MTdT-evo-mediated dsDNA extension with 2′-F-CTP and the combination of 1 mM MnCl_2_ and 10 mM Mg(OAc)_2_ was optimal for the MTdT-evo-mediated dsDNA extension with faCTP. These results suggested that the activities of TdTs for dsDNA extension with 2′-modified NTPs could also be effectively improved by optimizing the metal ions supplemented into the reaction solution.

### Activities of different TdTs for XNA extension with (d)NTPs containing a natural or unnatural nucleobase

**Figure 5.**
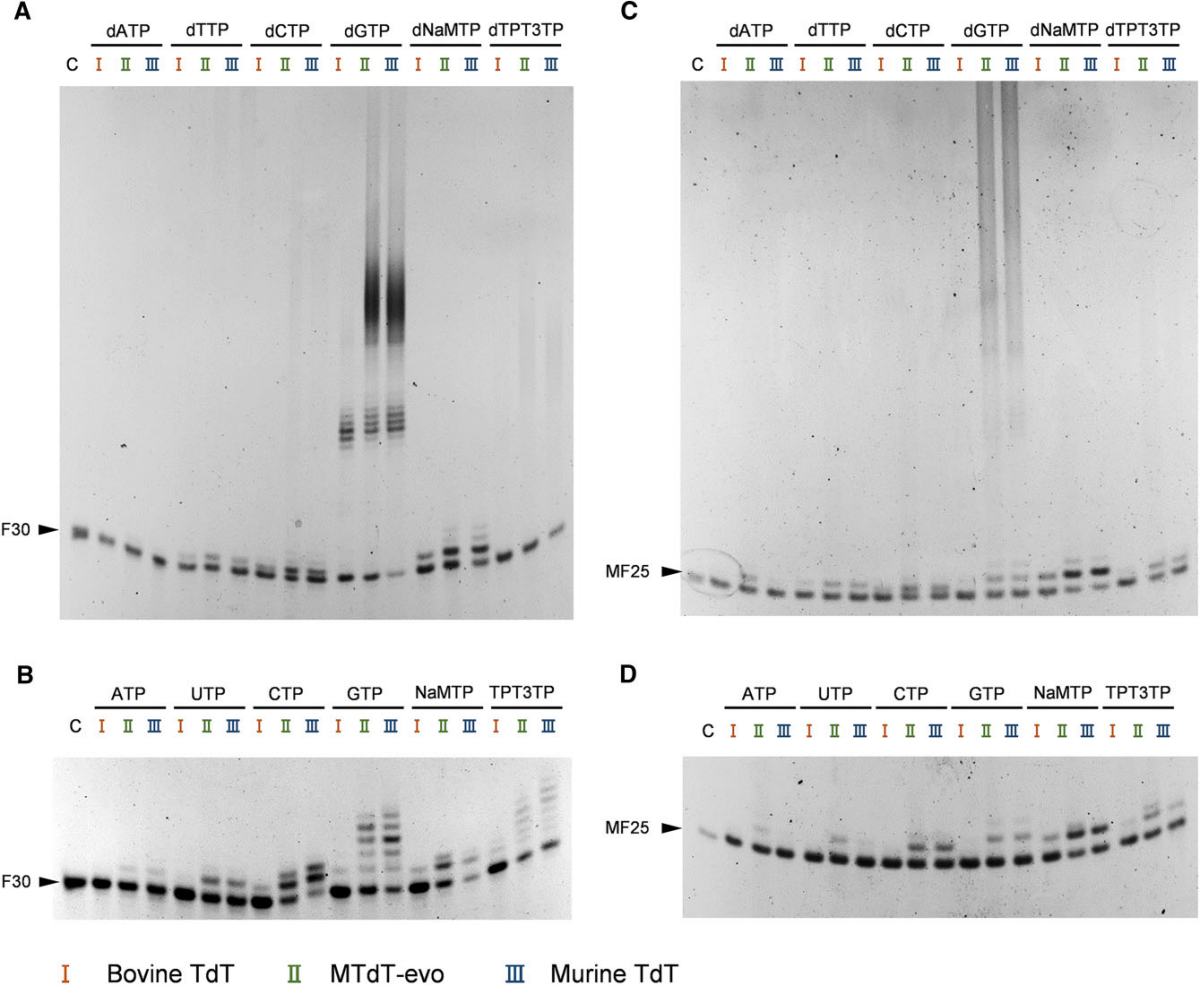
TdT-mediated extension of ssXNAs with (d)NTPs containing a natural or unnatural nucleobase. (**A**) Extension of single-stranded 2′-F-DNA F30 with dNTPs containing a natural or unnatural nucleobase. (**B**) Extension of single-stranded 2′-F-DNA F30 with NTPs containing a natural or unnatural nucleobase. (**C**) Extension of single-stranded 2′-OMe/F-DNA MF25 with dNTPs containing a natural or unnatural nucleobase. (**D**) Extension of single-stranded 2′-OMe/F-DNA MF25 with NTPs containing a natural or unnatural nucleobase. C, control (without the addition of TdT); I, extension by bovine TdT; II, extension by MTdT-evo; III, extension by murine TdT.

After the exploration of the three TdTs' activities to extend ssDNA and dsDNA strands with 2′-modified NTPs, we next investigated their activities to extend different single-stranded XNA (ssXNA) primers with (d)NTPs, which could be useful for the synthesis of XNA–DNA and XNA–RNA chimeric molecules and labelling of XNAs. Primer extension experiments were carried out with 2′-F-DNA primer F30 and 2′-OMe/F-DNA primer MF25 (Supplementary Table S1). These ssXNA primers were extended with different (d)NTPs containing a natural nucleobase or an unnatural nucleobase NaM or TPT3 (Figure 1B) using different TdTs in the presence of 1 mM CoCl_2_. Denaturing PAGE analysis of the products revealed that 2′-F-DNA primer F30 could be extended with most of the tested (d)NTPs, even those containing the unnatural nucleobase NaM or TPT3, by some or all of the TdTs, and the extension with dGTP was the most efficient for all TdTs (Figure 5A, B). 2′-OMe/F-DNA primer MF25 could also be extended with most of the tested (d)NTPs by some or all of the TdTs, although the extension efficiencies were generally lower than those of the 2′-F-DNA primer (Figure 5C, D). However, extension was barely observed for the 2′-OMe-DNA primer with any of the (d)NTPs (data not shown), probably due to large steric hindrances between the amino acid residues in the TdTs and the 2′-OMe groups of the nucleotides in the 3′ end of the primer. Notably, in most cases, MTdT-evo and murine TdT extended the XNA primers more efficiently than bovine TdT.

### 3′-End labelling of 2′-F-DNA by MTdT-evo-mediated synthesis of G-quadruplex-containing oligonucleotide tails

**Figure 6.**
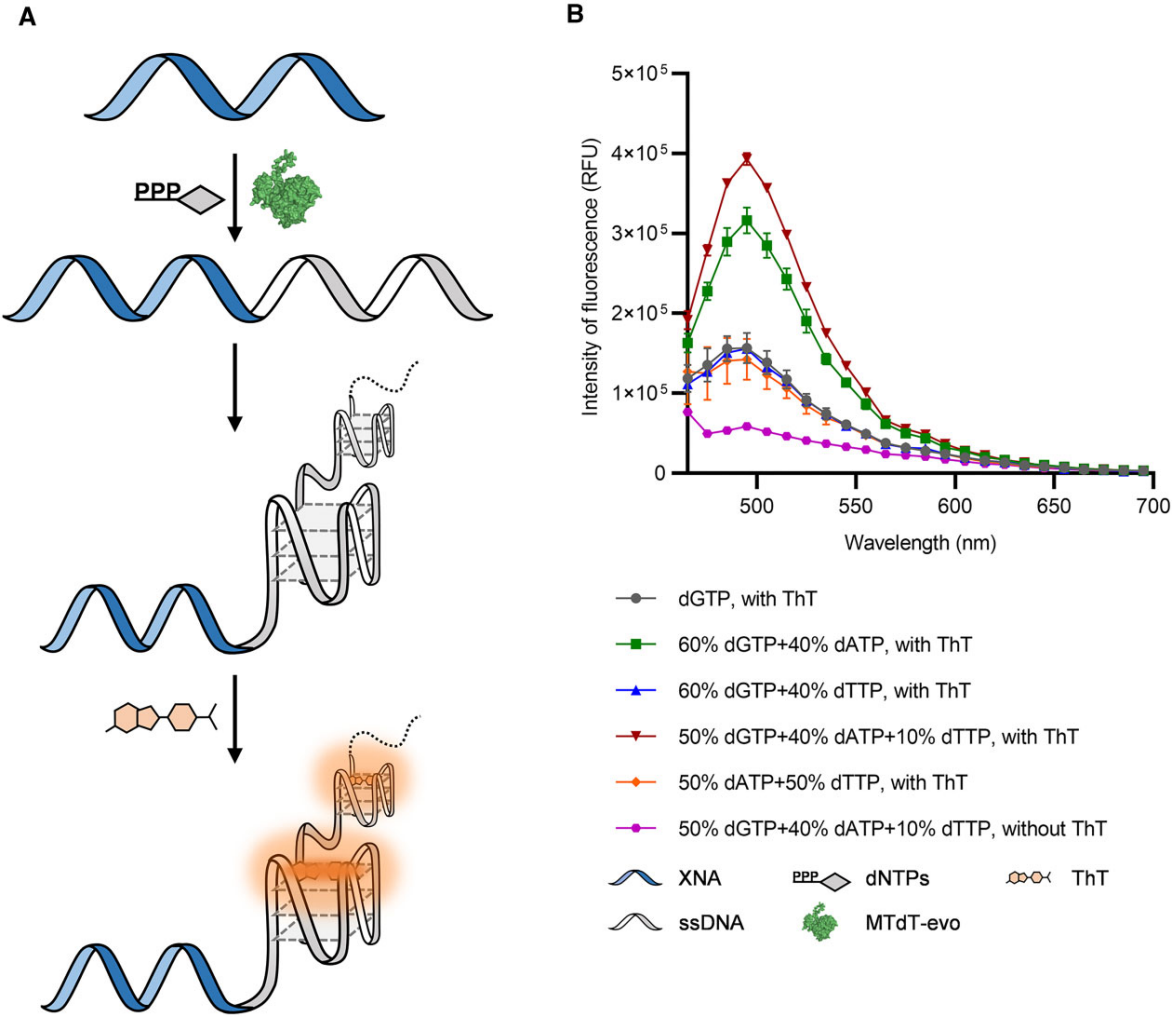
Fluorescent labelling of 2′-F-DNA by MTdT-evo-synthesized G-quadruplex-containing tails. (**A**) Scheme of the experiment. (**B**) Fluorescence emission spectra of 2′-F-DNAs labelled with different DNA oligonucleotide tails upon incubation with ThT. The DNA oligonucleotide tails were produced by extending 2′-F-DNA F30 with MTdT-evo and different combinations of dNTPs. Error bars indicate standard deviations from the means (*n* = 3).

G-quadruplexes have been found to be capable of binding to certain fluorogenic dyes which leads to a large increase in fluorescence (71). This allows the development of a series of G-quadruplex-based fluorescent sensors (72–74). Moreover, the binding of G-rich sequences and ThT dye has been successfully applied in real-time fluorescent detection of TdT activity (46). Given that a 2′-F-DNA primer could be well extended with dNTPs, especially dGTP, by TdTs, we tried to establish a method for fluorescent labelling of 2′-F-DNA via TdT-mediated synthesis of 3′ tails containing G-quadruplexes that can bind to ThT. A 200 nM concentration of 2′-F-DNA primer F30 was extended by mixing with different combinations of dNTPs (100 μM in total), including dGTP only, 60% dGTP + 40% dATP, 60% dGTP + 40% dTTP and 50% dGTP + 40% dATP + 10% dTTP (a reported optimal combination for the generation of fluorescence signal in TdT-mediated DNA labelling) (46), 1 mM CoCl_2_ and 1 μM MTdT-evo in 1× TdT buffer and incubating at 37°C for 30 min. The extension products were mixed with 0.1 mM ThT and the fluorescence emission spectra were collected over the range of 465–695 nm. As shown in Figure 6, the extension products with 50% dGTP + 40% dATP + 10% dTTP and 60% dGTP + 40% dTTP showed significant increases in fluorescence at 485 nm upon mixing with ThT, indicating the successful formation of G-quadruplex-containing tails that could bind to ThT. The fluorescence increase for the extension product with 50% dGTP + 40% dATP + 10% dTTP was the highest, suggesting that this combination of dNTPs was optimal for the fluorescent labelling of 2′-F-DNA. Clearly, it is feasible to fluorescently label 2′-F-DNA via MTdT-evo-mediated synthesis of 3′ tails containing G-quadruplexes.

### 3′-End labelling of XNAs by TdT-mediated incorporation of nucleotides containing a functionalized nucleobase

**Figure 7.**
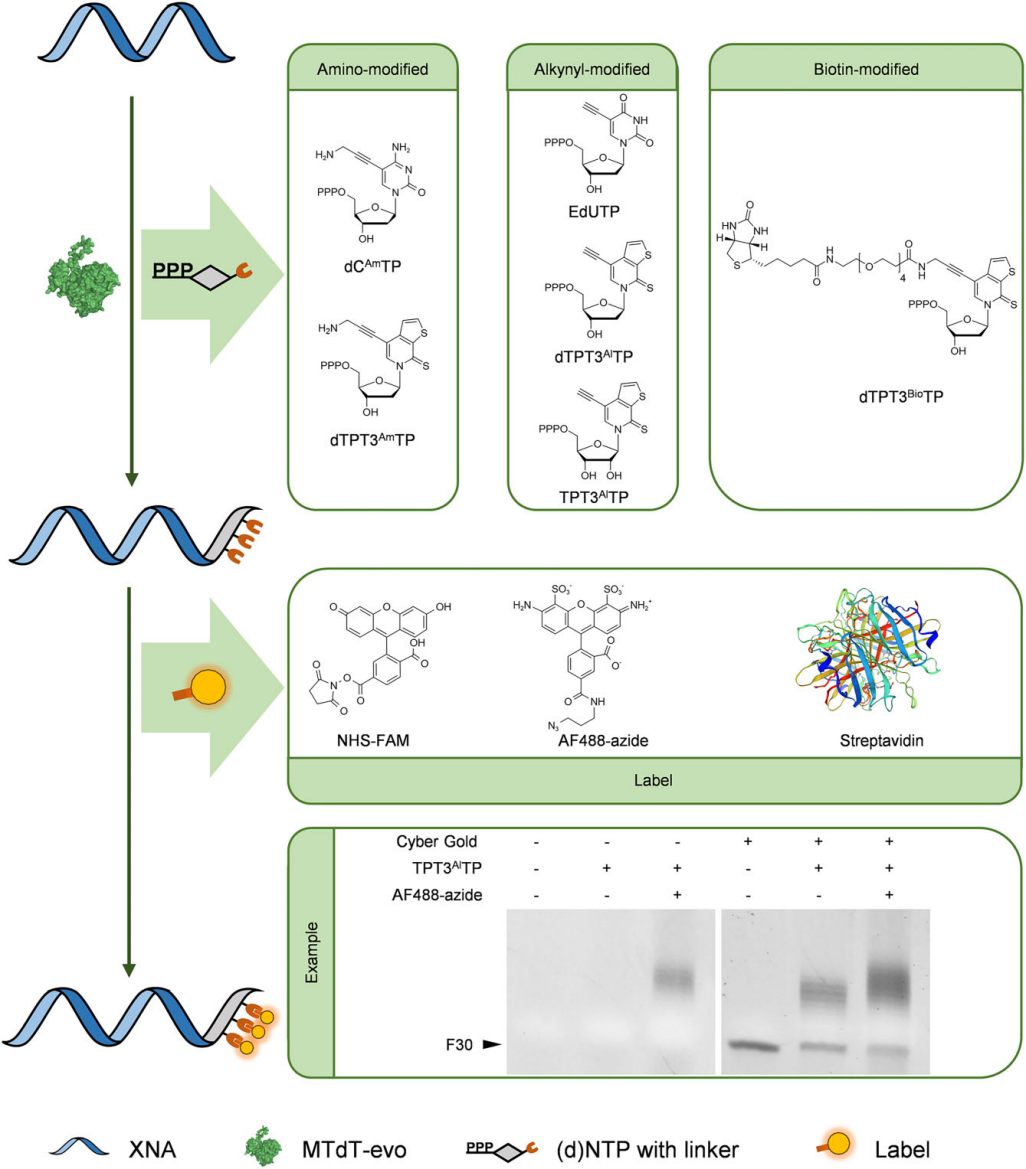
3′-End labelling of XNAs by TdT-mediated incorporation of nucleotides containing a functionalized nucleobase. XNAs can be labelled with various molecules by TdT-mediated incorporation of (d)NTPs containing an amino-, alkynyl- or biotinyl-modified nucleobase and subsequent coupling with NHS- or azide-conjugated molecules, exemplified by NHS-FAM or AF488-azide, or binding with SA or SA conjugates, respectively. The 3′-end labelling of 2′-F-DNA F30 by MTdT-evo-mediated incorporation of TPT3^Al^TP and subsequent coupling of TPT3^Al^ with AF488-azide via click reaction is shown as an example.

Besides fluorescent labels based on G-quadruplexes, other molecules or functionalities may also need to be attached to XNA molecules for different application scenarios. Functionalized nucleobases have been widely used for coupling DNA and RNA with other molecules (37,75). Previously, we have demonstrated the efficient and orthogonal 3′ labelling of ssDNAs with nucleobase-functionalized dTPT3TP derivatives by TdT (61). Inspired by the effiecient activities of TdTs for extending 2′-F-DNA and 2′-OMe/F-DNA strands with (d)NTPs, we further explored the capabilities of TdTs for extending these XNAs by incorporating (d)NTPs containing a functionalized natural or unnatural nucleobase into their 3′ ends (Figure 7). 2′-F-DNA primer F30 and 2′-OMe/F-DNA primer MF25 were extended with dC^Am^TP, EdUTP, alkynyl-modified dTPT3TP (dTPT3^Al^TP), amino-modified dTPT3TP (dTPT3^Am^TP), dTPT3TP with a biotinylated linker (dTPT3^Bio^TP) or alkyne-modified TPT3TP (TPT3^Al^TP) by bovine TdT, MTdT-evo or murine TdT. The extension products were also analysed by denaturing PAGE. As shown in Supplementary Figure S18, a majority of the functionalized (d)NTPs could be incorporated into the 3′ ends of the 2′-F-DNA primer F30 and the 2′-OMe/F-DNA primer MF25 by some or all three TdTs. In general, MTdT-evo exhibited the highest efficiencies for the incorporation of these (d)NTPs. Clearly, among these three TdTs, MTdT-evo is optimal for the labelling of 2′-F-DNA and 2′-OMe/F-DNA with functionalized (d)NTPs. The time courses of MTdT-evo-mediated ssXNA extension with different functionalized (d)NTPs were further investigated. Primers F30 and MF25 were extended with 10 μM of one of the functionalized (d)NTPs by MTdT-evo in the presence of 1 mM CoCl_2_ for different reaction times, and the products were analysed with denaturing PAGE gels. As shown in Supplementary Figure S19A and B, for the extension of F30 with dC^Am^TP or TPT3^Al^TP and the extension of MF25 with dC^Am^TP, dTPT3^Am^TP or TPT3^Al^TP, increasing amounts of primer were extended and increasing numbers of nucleotides were incorporated along with the increase of the reaction time in the tested range (5–80 min). For the extension of F30 with dTPT3^Am^TP, an increasing amount of primer was extended and increasing numbers of nucleotides were incorporated along with the increase of the reaction time in the range of 5–20 min, and a further increase of the reaction time to >20 min did not lead to much improvement of the extension, suggesting that 20 min is optimal for this extension. For the extension of both F30 and MF25 with EdUTP, dTPT3^Al^TP or dTPT3^Bio^TP, in the tested range (5–80 min), a longer reaction time did not lead to significant improvement of the extension. These results suggested that it is feasible to label XNAs with different functional groups by incorporating (d)NTPs with a functionalized nucleobase using TdTs and in some cases the number of the incorporated nucleotides is controllable to meet the requirements of different applications. Taking advantage of these functional groups, various molecules can then be attached to the XNAs (Figure 7). In particular, the XNAs with 3′-end nucleotides containing an amino- or alkynyl-modified nucleobase can be coupled with NHS- or azide-conjugated molecules, exemplified by NHS-FAM or AF488-azide, and the XNAs with 3′-end nucleotides containing a biotinyl-modified nucleobase can bind with streptavidin (SA) or SA conjugates. As an example, 3′-end fluorescent labelling of 2′-F-DNA F30 was carried out by MTdT-evo-mediated incorporation of TPT3^Al^TP and subsequent coupling of TPT3^Al^ with AF488-azide via click chemistry. F30 at 100 nM was mixed with 10 μM TPT3^Al^TP, 1 mM CoCl_2_ and 0.1 μM MTdT-evo in 1× TdT buffer and incubated at 37°C for 30 min. The extension product was purified and incubated with 100 μM AF488-azide, 100 μM CuSO_4_, 500 μM THPTA and 2.5 mM ascorbic acid, at 37°C for 30 min. The coupling product was analysed by denaturing PAGE. The successful coupling of AF488-azide and F30 extended with TPT3^Al^TP was proven by the appearance of fluorescent bands at correct positions on a denaturing PAGE gel before the gel was stained with Cyber gold, suggesting the feasibility of this strategy for labelling XNAs with desired molecules.

### Construction of a DNA–FANA chimeric hydrogel based on TdT’s capability for efficient FANA synthesis

**Figure 8.**
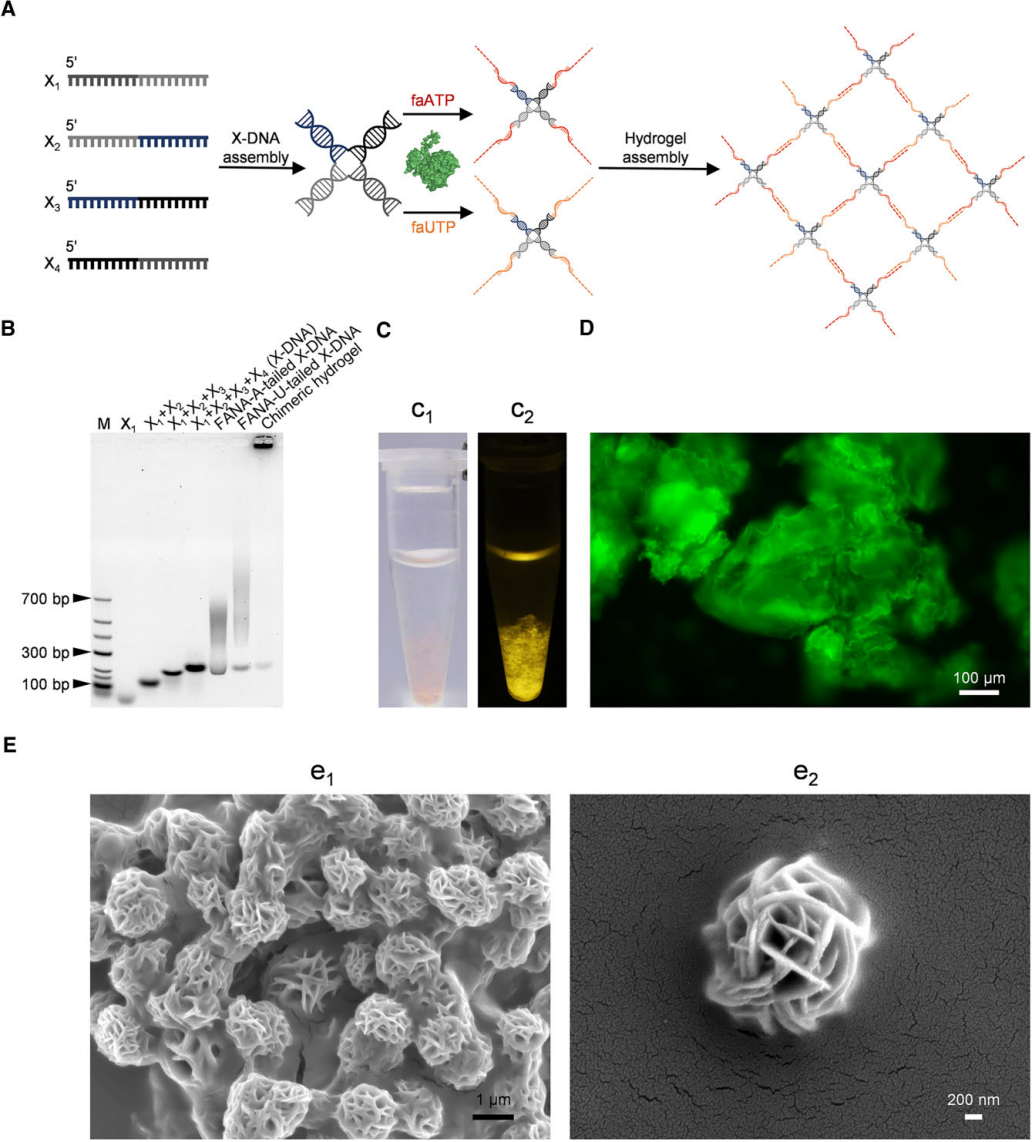
MTdT-evo-facilitated construction and characterization of a DNA–FANA chimeric hydrogel. (**A**) Construction scheme of the DNA–FANA chimeric hydrogel. (**B**) Gel analysis of the products for the assembly of X-DNA and construction of the DNA–FANA chimeric hydrogel. M, low molecular weight ladder. (**C**) Morphology of the DNA–FANA chimeric hydrogel. The hydrogel was stained with Cyber gold. c_1_, under natural light; c_2_, under 470 nm blue light. (**D**) Fluorescence microscope image of the DNA–FANA chimeric hydrogel. The hydrogel was stained with Cyber gold. (**E**) SEM images of the DNA–FANA chimeric hydrogel. The hydrogel was dehydrated and coated with gold before SEM imaging with different magnifications (e_1_, low magnification and e_2_, high magnification).

To explore the application of TdTs' activity for XNA synthesis in the development of novel biomaterials, we attempted to construct a DNA–FANA chimeric hydrogel, taking advantage of MTdT-evo's capability for FANA synthesis to prepare structural components (Figure 8A). Four DNA oligonucleotides, X_1_, X_2_, X_3_ and X_4_ (Supplementary Table S1), were first annealed to assemble an X-shaped DNA (X-DNA), which served as the initiator for the polymerization of faNTPs mediated by MTdT-evo. The X-DNA was then extended by mixing it with 5 mM faATP or faUTP, 10 mM Mg(OAc)_2_, 1 mM MnCl_2_ and 10 μM MTdT-evo in 1× TdT buffer, and incubating at 37°C for 12 h. The Mg(OAc)_2_ and MnCl_2_ were added based on the optimization results of the reaction conditions for dsDNA extension described above. The extension products were then concentrated, mixed and annealed to produce the DNA–FANA chimeric hydrogel. The products of each step were analysed with a 1.5% agarose gel. As shown in Figure 8B, for the preparation of the X-DNA, sequential addition of the DNA oligonucleotides led to a gradual decrease in the migration speed of the products, indicating a successful assembly of these oligonucleotides. Much higher bands were observed for the X-DNA extension products, suggesting the efficient incorporation of faATP and faUTP onto the 3′ ends of the X-DNA. The assembly product of the X-DNAs extended with faATP and faUTP exhibited no obvious migration in the gel, indicating the formation of the DNA–FANA chimeric hydrogel with ultra-high molecular weight. The DNA–FANA chimeric hydrogel appeared as a flocculent precipitate in the aqueous solution (Figure 8C, c_1_), which was somehow different from the reported hydrogel of pure DNA prepared with TdT using a similar procedure (48). After staining with Cyber gold, the DNA–FANA chimeric hydrogel emitted bright fluorescence under 470 nm blue light (Figure 8C, c_2_), reflecting its composition of nucleic acids. Observation with a fluorescence microscope demonstrated that the DNA–FANA chimeric hydrogel had a loose microstructure with a large specific surface area (Figure 8D), while imaging with an SEM revealed nanoflower structures in the lyophilized DNA–FANA chimeric hydrogel (Figure 8E). Similar structures have also been observed for DNA hydrogels reported in other studies (76–80). These structural features implied the potential of use of this DNA–FANA chimeric hydrogel in various applications, including encapsulation of enzymes, capture and culture of cells and construction of novel biosensors (81–84). Based on these results, it is clear that novel biomaterials containing XNA compositions can be conveniently prepared taking advantage of TdTs' ability for XNA synthesis.

## Conclusions

In summary, we first systematically explored bovine TdT, MTdT-evo and murine TdT for their activities to incorporate 2′-OMe-NTPs, 2′-F-NTPs and faNTPs into the 3′ end of an ssDNA primer. Among all TdTs, MTdT-evo exhibited the highest efficiency for ssDNA extension with most of the 2′-modified NTPs. The TdT-mediated template-independent synthesis of FANA was found to be generally more efficient than those of 2′-F-DNA and 2′-OMe-DNA. The reaction conditions for MTdT-evo-mediated incorporation of different 2′-modified NTPs were then optimized. The effect of the 3′-end nucleotide of the ssDNA primer on MTdT-evo-mediated incorporation of 2′-modified NTPs was also demonstrated. Steady-state kinetic experiments were carried out to quantitatively characterize TdTs for their activities to incorporate different 2′-modified NTPs. The results suggested that for the incorporation of a single nucleotide, among the three TdTs, MTdT-evo and murine TdT are more efficient for 2′-OMe-NTPs, murine TdT is more efficient for 2′-F-NTPs, and MTdT-evo and murine TdT are more efficient for faNTPs. In most cases, faNTP is more efficiently incorporated than the corresponding 2′-F-NTP, and 2′-F-NTP is more efficiently incorporated than the corresponding 2′-OMe-NTP. Strikingly, the catalytic efficiencies of all the three TdTs for the incorporation of faNTPs were found to be comparable with or even higher than those for the incorporation of corresponding dNTPs, and the catalytic efficiencies of murine TdT for the incorporation of 2′-F-NTPs were found to be close to or even higher than those for the incorporation of corresponding dNTPs. Based on MTdT-evo's ability to incorporate 2′-modified NTPs into the 3′ end of an ssDNA molecule, we then developed a convenient method for the end protection of ssDNAs against exonuclease degradation and 2′-OMe-CTP was found to be the best 2′-OMe-NTP to be used in this method. Next, the TdTs' activities for the extension of dsDNA with 2′-modified NTPs were explored to find that most of the 2′-modified NTPs could be incorporated into the 3′ ends of dsDNA by different TdTs with varied efficiencies. The reaction conditions were also optimized using MTdT-evo-mediated incorporation of 2′-modified CTPs as a model. The TdTs' activities for extending XNAs with (d)NTPs containing a natural or unnatural nucleobase or a functionalized nucleobase derivative were then explored and demonstrated. Based on these activities, methods for labelling XNAs by TdT-mediated synthesis of G-quadruplex-containing tails or incorporation of nucleotides containing a functionalized nucleobase have been established. These methods may find a wide use in many application scenarios of XNAs, including development of XNA-based probes and biosensors, preparation of XNA–small molecule/macromolecule conjugates and construction of XNA-scaffolded nanostructures and devices. Finally, a DNA–FANA chimeric hydrogel was successfully developed and characterized based on MTdT-evo's excellent activity for template-independent synthesis of FANA, suggesting the great potential of TdT-mediated XNA synthesis in the construction of novel biomaterials.

## Supplementary Material

gkae691_Supplemental_File

## Data Availability

The data underlying this article are available in the article and in its online supplementary data.
