# Supplementary material for: Template-independent synthesis and 3′-end labelling of 2′-modified oligonucleotides with terminal deoxynucleotidyl transferases
Source: Nucleic Acids Res. 2024 Aug 16;52(17):10085–101. doi: 10.1093/nar/gkae691 (PMC11417362; doi:10.1093/nar/gkae691)
Supplement: gkae691_Supplemental_File [file gkae691_supplemental_file.pdf]

Supporting Information

Template-Independent Synthesis and 3'-End Labelling of 2'-Modified Oligonucleotides with Terminal Deoxynucleotidyl Transferases

Leping Sun[1,†], Yuming Xiang[1,†], Yuhui Du[1,†], Yangming Wang[1], Jiezhao Ma[1], Yaxin Wang[1], Xueting Wang[1], Guangyuan Wang[1], and Tingjian Chen[1,*]

1. MOE International Joint Research Laboratory on Synthetic Biology and Medicines, School of Biology and Biological Engineering, South China University of Technology, 510006, Guangzhou, China.

*Corresponding author: chentj@scut.edu.cn

[†]The first three authors should be regarded as Joint First Authors.

Supplementary Figures

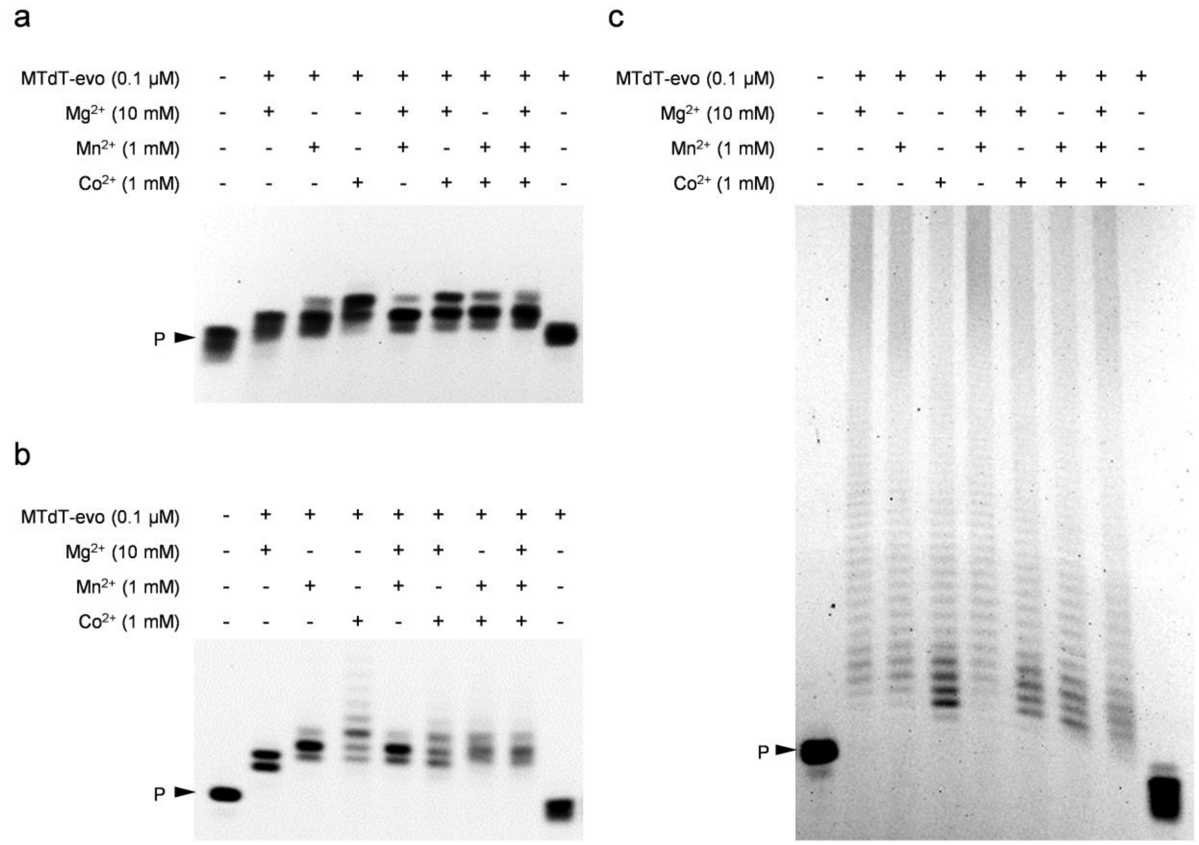

Figure S1. Effects of metal ions on MTdT-evo-mediated primer extension with 2'-modified NTPs. a. Extension with 2'-OMe-CTP. b. Extension with 2'-F-CTP. c. Extension with faCTP. For each reaction, 100 nM primer FAM-18G was mixed with 10 μM one of 2'-modified CTPs and 0.1 μM MTdT-evo in 1× TdT buffer with 1 mM $CoCl_2$, 1 mM $MnCl_2$, 10 mM $Mg(OAc)_2$, or one of their combinations, and incubated at 37 °C for 30 min. The products were analyzed with 20% denaturing PAGE gels supplemented with 8 M urea. P: primer FAM-18G.

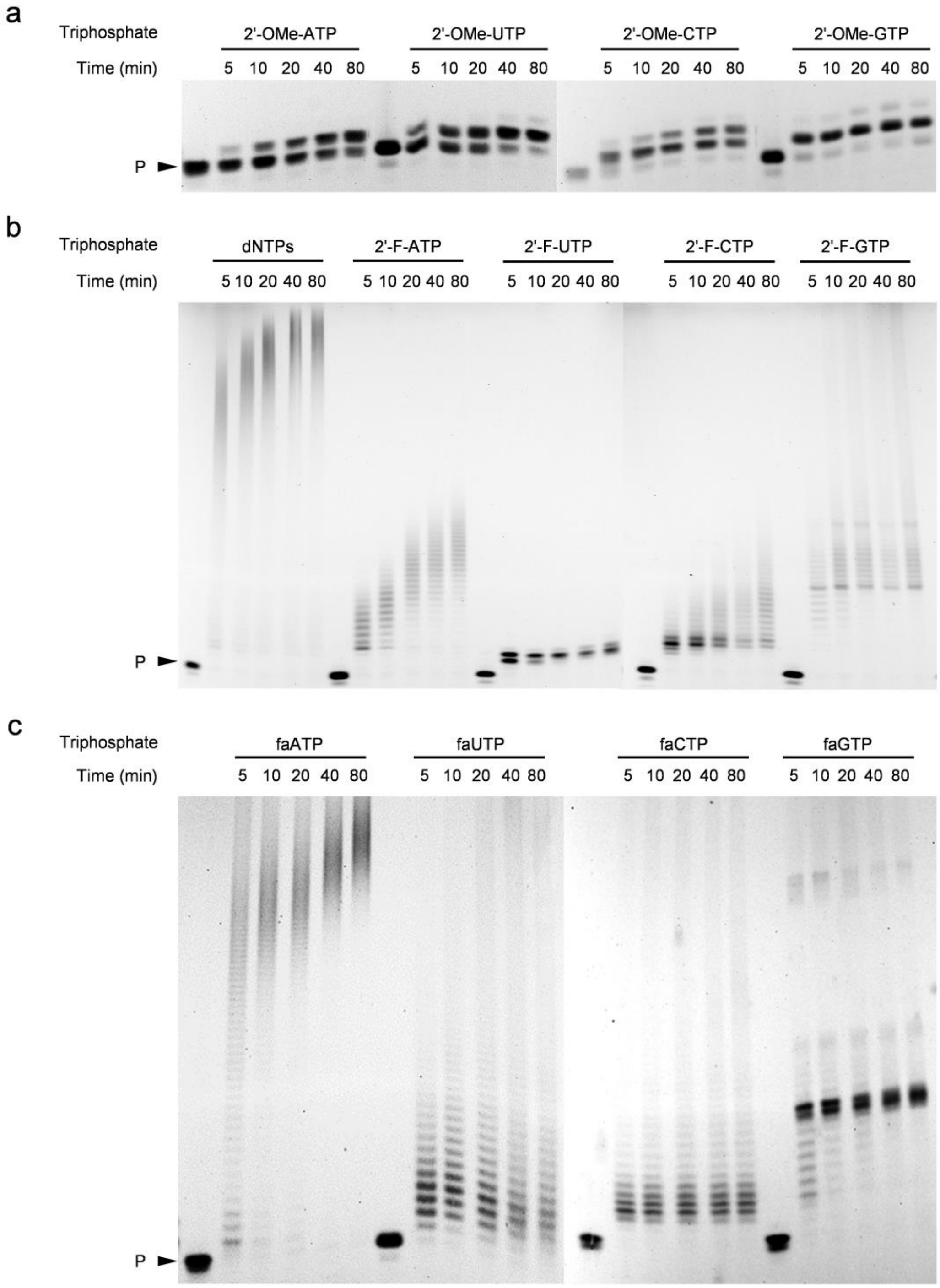

Figure S2. Time courses of MTdT-evo-mediated ssDNA extension with different nucleoside triphosphates. a. Extension with 2'-OMe-NTPs. b. Extension with dNTPs or 2'-F-NTPs. c. Extension with faNTPs. For each reaction, 100 nM primer FAM-18G was mixed with 10 μM one of 2'-modified NTPs, or 2.5 μM each of dNTPs, 1 mM $CoCl_2$, and 0.1 μM MTdT-evo in 1× TdT buffer and incubated at 37 °C for 5-80 min. The products were analyzed with 20% denaturing PAGE gels supplemented with 8 M urea. P: primer FAM-18G.

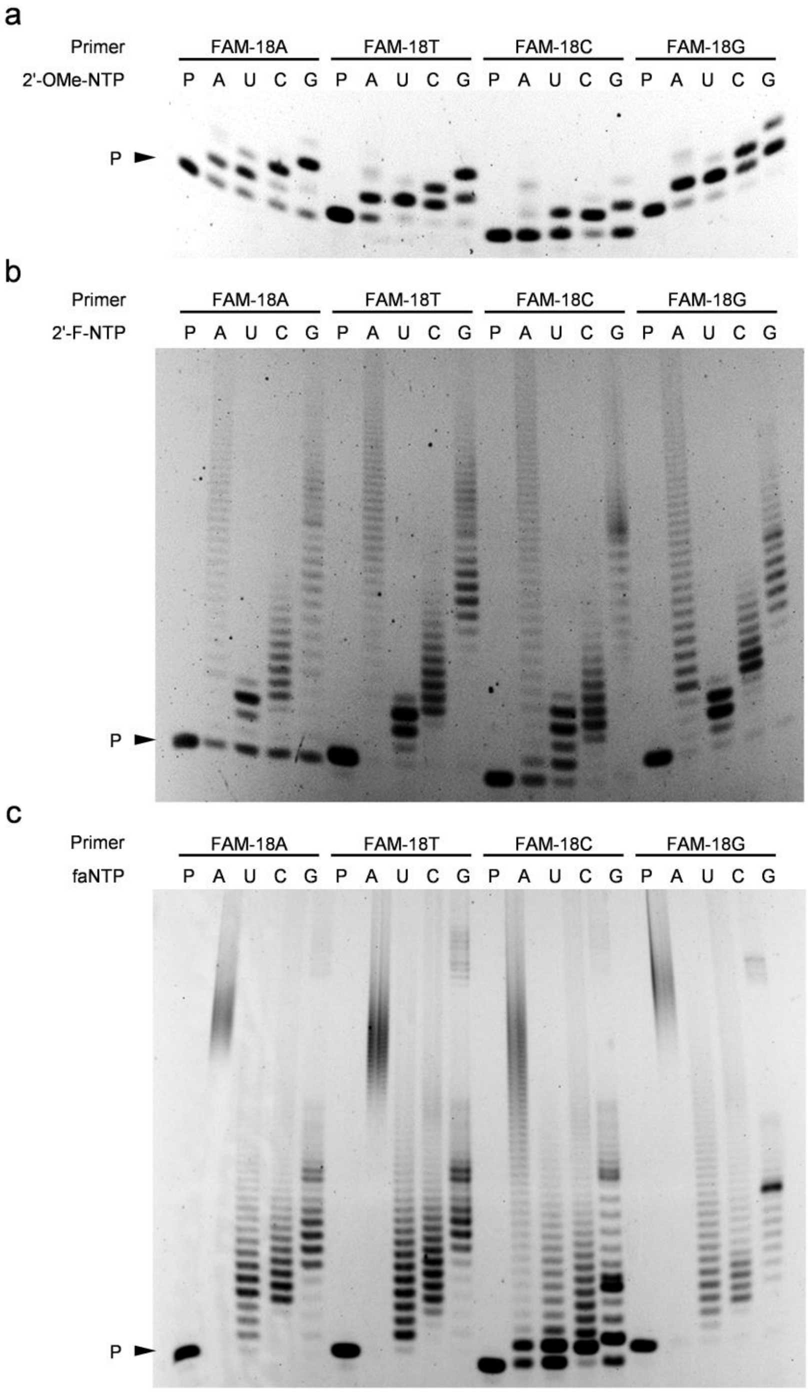

Figure S3. Effects of 3'-end nucleotides on MTdT-evo-mediated primer extension with 2'-modified NTPs. a. Extension with 2'-OMe-NTPs. b. Extension with 2'-F-NTPs. c. Extension with faNTPs. For each reaction, 100 nM primer was mixed with 10 μM one of 2'-modified NTPs, 1 mM $CoCl_2$, and 0.1 μM MTdT-evo in 1× TdT buffer and incubated at 37 °C for 30 min. The products were analyzed with 20% denaturing PAGE gels supplemented with 8 M urea. P: corresponding primer.

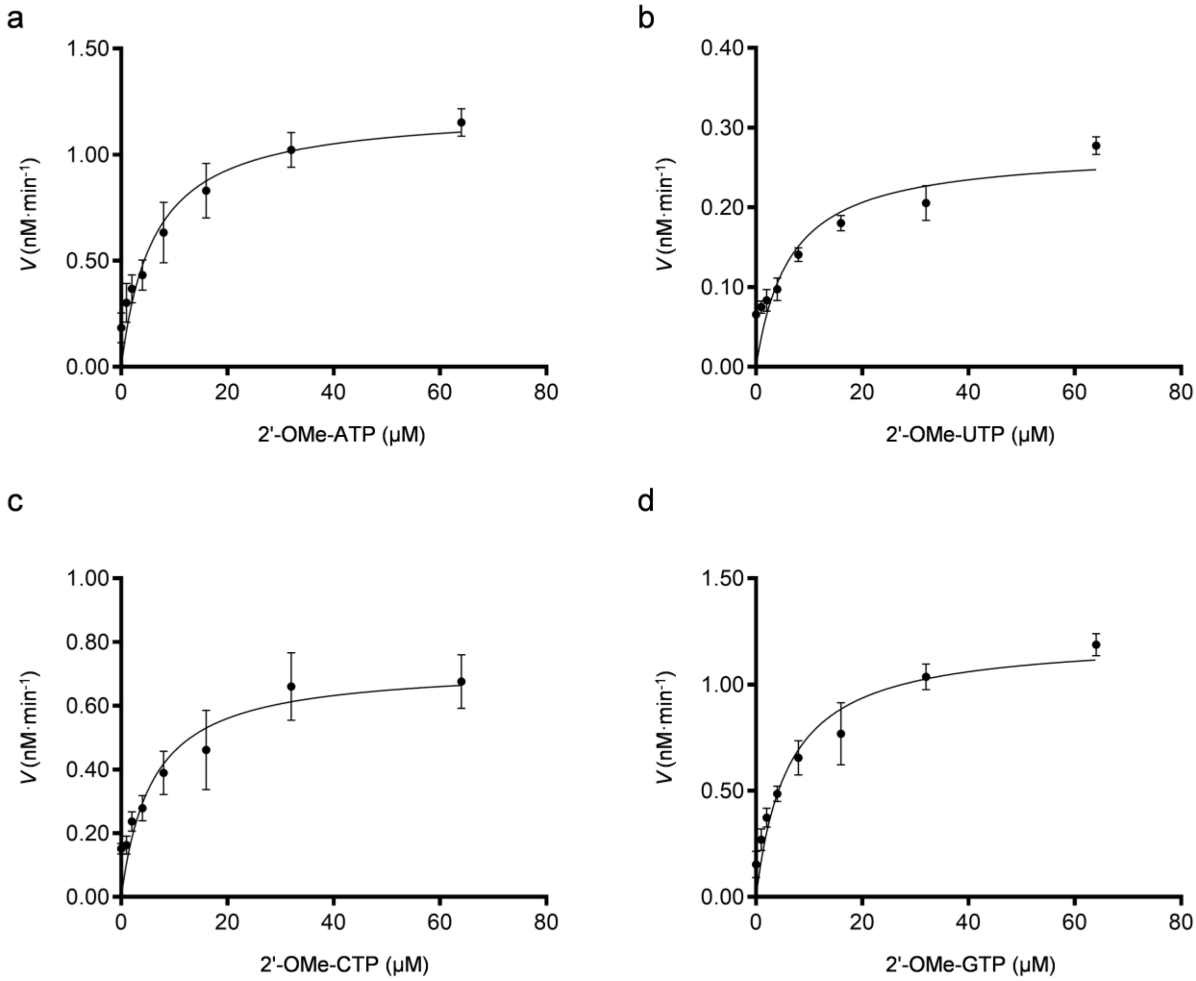

Figure S4. Michaelis-Menten plots for bovine TdT-mediated incorporation of 2'-OMe-NTPs. a. Incorporation of 2'-OMe-ATP. 100 nM primer FAM-18G (Table S1) was mixed with 0-64 μM 2'-OMe-ATP, 10 mM $Mg(OAc)_2$, and 10 nM bovine TdT in 1× TdT buffer and incubated at 37 °C for 20 min. b. Incorporation of 2'-OMe-UTP. 100 nM primer FAM-18G was mixed with 0-64 μM 2'-OMe-UTP, 10 mM $Mg(OAc)_2$, and 60 nM bovine TdT in 1× TdT buffer and incubated at 37 °C for 82 min. c. Incorporation of 2'-OMe-CTP. 100 nM primer FAM-18G was mixed with 0-64 μM 2'-OMe-CTP, 10 mM $Mg(OAc)_2$, and 30 nM bovine TdT in 1× TdT buffer and incubated at 37 °C for 30 min. d. Incorporation of 2'-OMe-GTP. 100 nM primer FAM-18G was mixed with 0-64 μM 2'-OMe-GTP, 10 mM $Mg(OAc)_2$, and 30 nM bovine TdT in 1× TdT buffer and incubated at 37 °C for 30 min. The products were analyzed with 20% denaturing PAGE gels supplemented with 8 M urea.

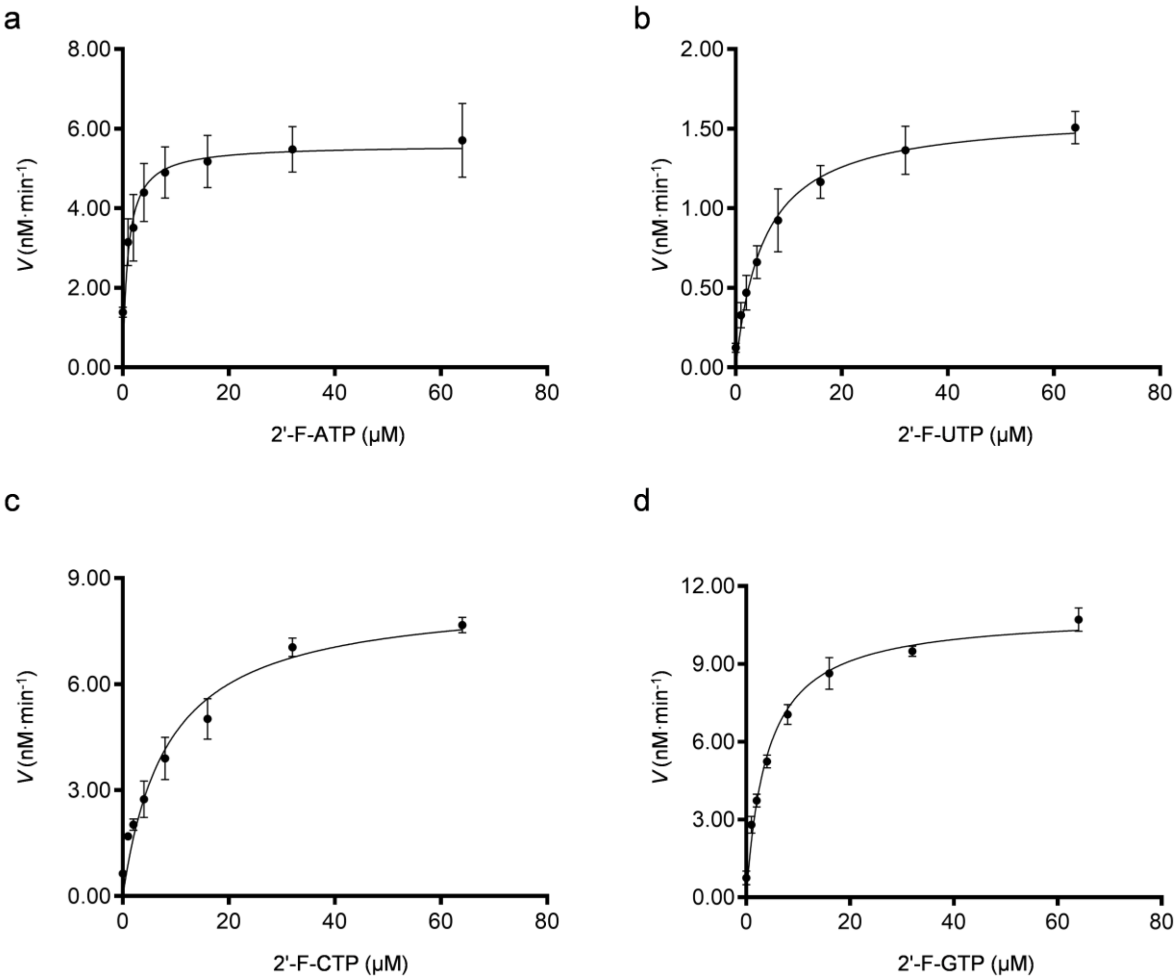

Figure S5. Michaelis-Menten plots for bovine TdT-mediated incorporation of 2'-F-NTPs. a. Incorporation of 2'-F-ATP. 100 nM primer FAM-18G was mixed with 0-64 μM 2'-F-ATP, 10 mM $Mg(OAc)_2$, and 10 nM bovine TdT in 1× TdT buffer and incubated at 37 °C for 5 min. b. Incorporation of 2'-F-UTP. 100 nM primer FAM-18G was mixed with 0-64 μM 2'-F-UTP, 10 mM $Mg(OAc)_2$, and 10 nM bovine TdT in 1× TdT buffer and incubated at 37 °C for 20 min. c. Incorporation of 2'-F-CTP. 100 nM primer FAM-18G was mixed with 0-64 μM 2'-F-CTP, 10 mM $Mg(OAc)_2$, and 10 nM bovine TdT in 1× TdT buffer and incubated at 37 °C for 6 min. d. Incorporation of 2'-F-GTP. 100 nM primer FAM-18G was mixed with 0-64 μM 2'-F-GTP, 10 mM $Mg(OAc)_2$, and 10 nM bovine TdT in 1× TdT buffer and incubated at 37 °C for 3 min. The products were analyzed with 20% denaturing PAGE gels supplemented with 8 M urea.

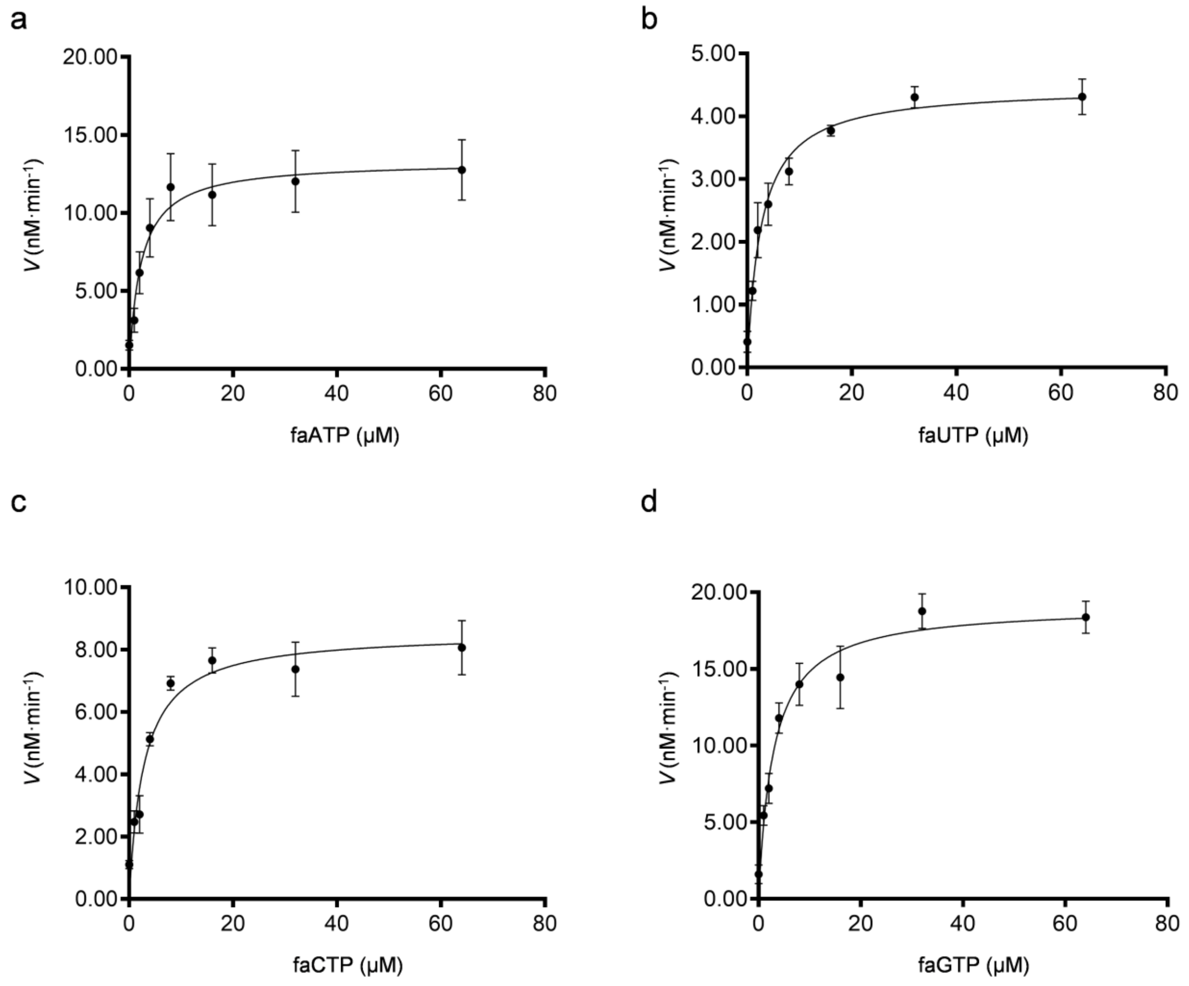

Figure S6. Michaelis-Menten plots for bovine TdT-mediated incorporation of faNTPs. a. Incorporation of faATP. 100 nM primer FAM-18G was mixed with 0-64 μM faATP, 10 mM $Mg(OAc)_2$, and 5 nM bovine TdT in 1× TdT buffer and incubated at 37 °C for 2 min. b. Incorporation of faUTP. 100 nM primer FAM-18G was mixed with 0-64 μM faUTP, 10 mM $Mg(OAc)_2$, and 5 nM bovine TdT in 1× TdT buffer and incubated at 37 °C for 6 min. c. Incorporation of faCTP. 100 nM primer FAM-18G was mixed with 0-64 μM faCTP, 10 mM $Mg(OAc)_2$, and 5 nM bovine TdT in 1× TdT buffer and incubated at 37 °C for 3.5 min. d. Incorporation of faGTP. 100 nM primer FAM-18G was mixed with 0-64 μM faGTP, 10 mM $Mg(OAc)_2$, and 5 nM bovine TdT in 1× TdT buffer and incubated at 37 °C for 2 min. The products were analyzed with 20% denaturing PAGE gels supplemented with 8 M urea.

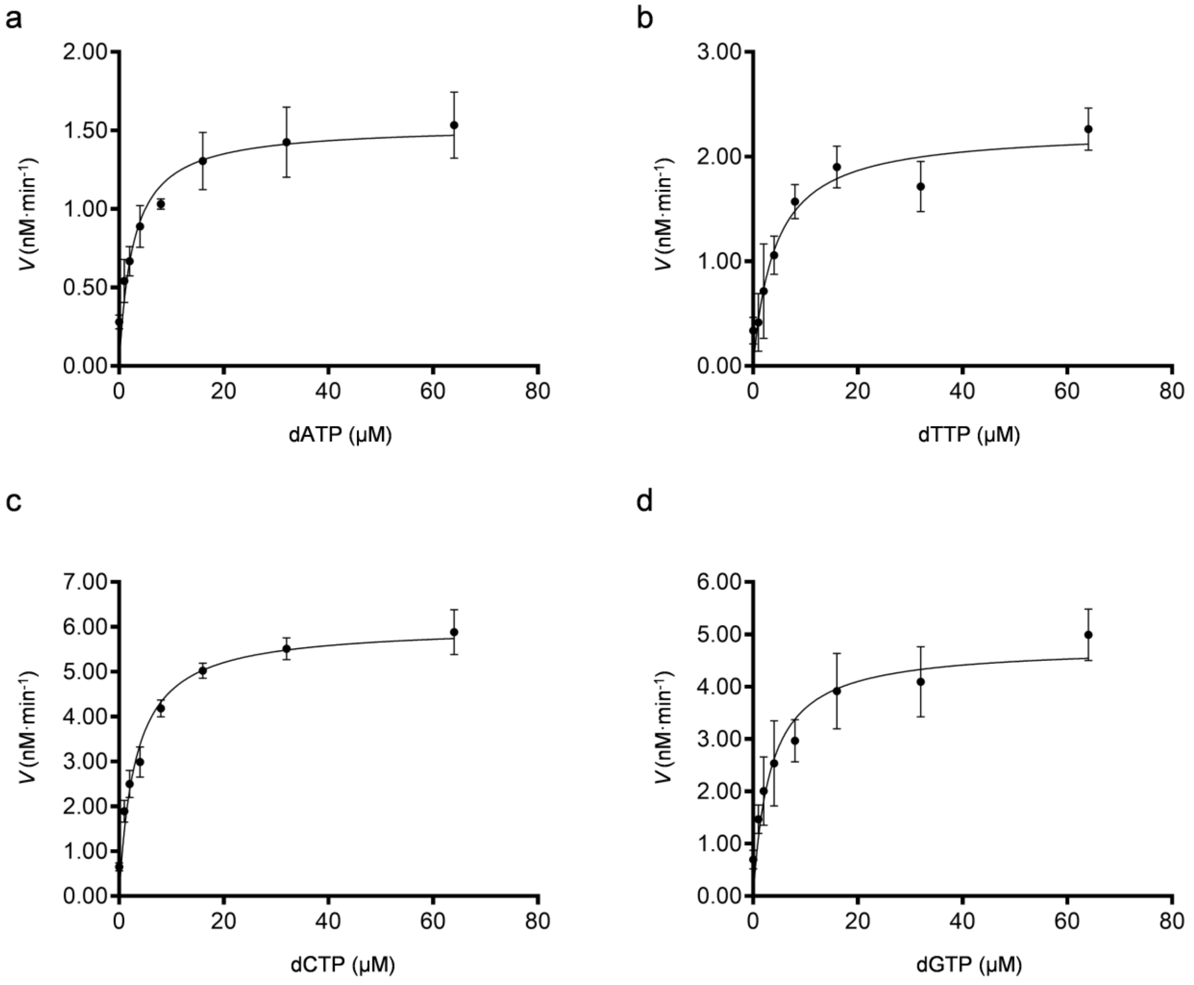

Figure S7. Michaelis-Menten plots for bovine TdT-mediated incorporation of dNTPs. a. Incorporation of dATP. 100 nM primer FAM-18G was mixed with 0-64 μM dATP, 10 mM $Mg(OAc)_2$, and 5 nM bovine TdT in 1× TdT buffer and incubated at 37 °C for 15 min. b. Incorporation of dTTP. 100 nM primer FAM-18G was mixed with 0-64 μM dTTP, 10 mM $Mg(OAc)_2$, and 5 nM bovine TdT in 1× TdT buffer and incubated at 37 °C for 10 min. c. Incorporation of dCTP. 100 nM primer FAM-18G was mixed with 0-64 μM dCTP, 10 mM $Mg(OAc)_2$, and 5 nM bovine TdT in 1× TdT buffer and incubated at 37 °C for 6 min. d. Incorporation of dGTP. 100 nM primer FAM-18G was mixed with 0-64 μM dGTP, 10 mM $Mg(OAc)_2$, and 5 nM bovine TdT in 1× TdT buffer and incubated at 37 °C for 6 min. The products were analyzed with 20% denaturing PAGE gels supplemented with 8 M urea.

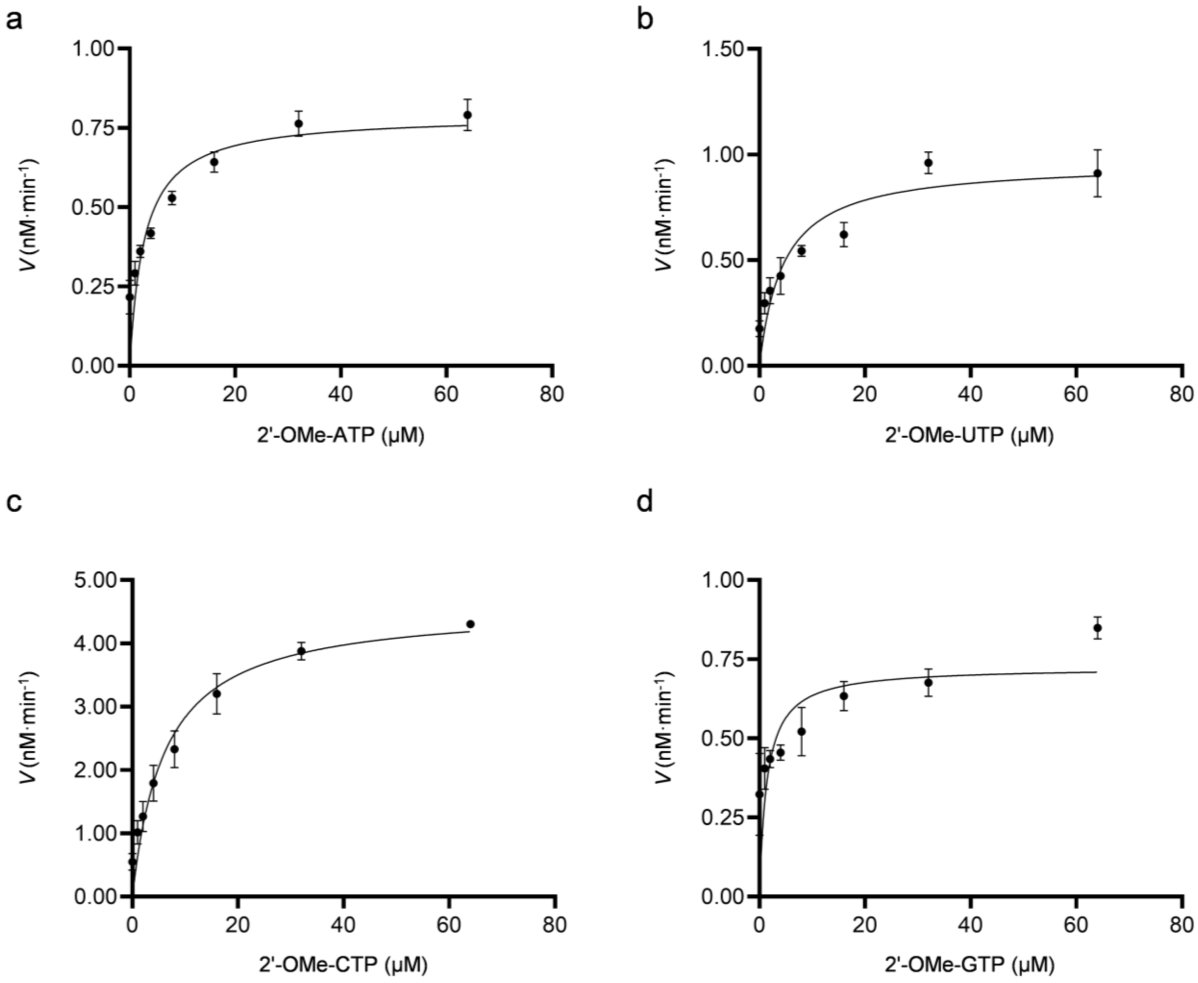

Figure S8. Michaelis-Menten plots for MTdT-evo-mediated incorporation of 2'-OMe-NTPs. a. Incorporation of 2'-OMe-ATP. 100 nM primer FAM-18G (Table S1) was mixed with 0-64 μM 2'-OMe-ATP, 10 mM $Mg(OAc)_2$, and 10 nM MTdT-evo in 1× TdT buffer and incubated at 37 °C for 30 min. b. Incorporation of 2'-OMe-UTP. 100 nM primer FAM-18G was mixed with 0-64 μM 2'-OMe-UTP, 10 mM $Mg(OAc)_2$, and 20 nM MTdT-evo in 1× TdT buffer and incubated at 37 °C for 30 min. c. Incorporation of 2'-OMe-CTP. 100 nM primer FAM-18G was mixed with 0-64 μM 2'-OMe-CTP, 10 mM $Mg(OAc)_2$, and 10 nM MTdT-evo in 1× TdT buffer and incubated at 37 °C for 10 min. d. Incorporation of 2'-OMe-GTP. 100 nM primer FAM-18G was mixed with 0-64 μM 2'-OMe-GTP, 10 mM $Mg(OAc)_2$, and 10 nM MTdT-evo in 1× TdT buffer and incubated at 37 °C for 30 min. The products were analyzed with 20% denaturing PAGE gels supplemented with 8 M urea.

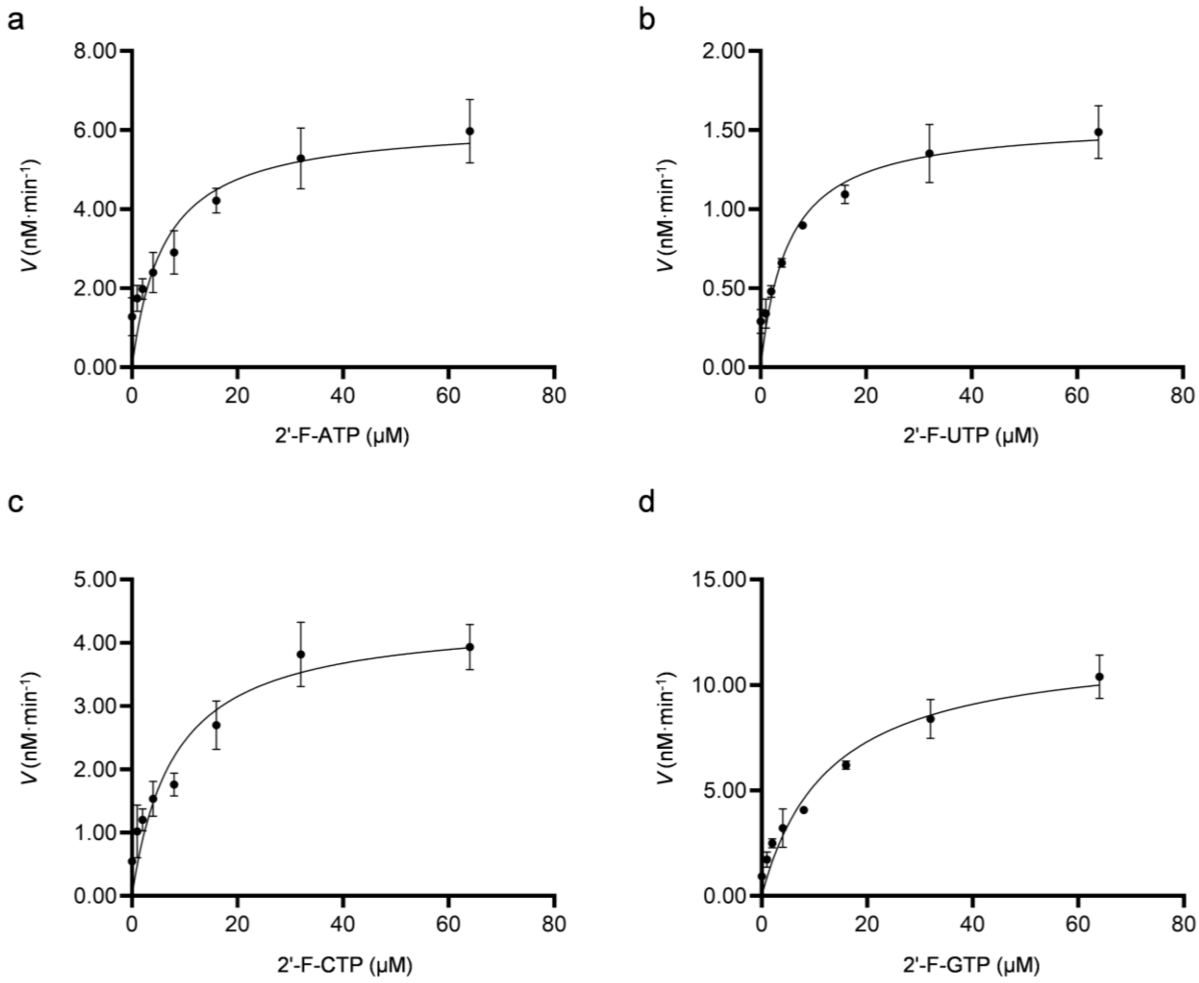

Figure S9. Michaelis-Menten plots for MTdT-evo-mediated incorporation of 2'-F-NTPs. a. Incorporation of 2'-F-ATP. 100 nM primer FAM-18G was mixed with 0-64 μM 2'-F-ATP, 10 mM $Mg(OAc)_2$, and 4 nM MTdT-evo in 1× TdT buffer and incubated at 37 °C for 4 min. b. Incorporation of 2'-F-UTP. 100 nM primer FAM-18G was mixed with 0-64 μM 2'-F-UTP, 10 mM $Mg(OAc)_2$, and 10 nM MTdT-evo in 1× TdT buffer and incubated at 37 °C for 16 min. c. Incorporation of 2'-F-CTP. 100 nM primer FAM-18G was mixed with 0-64 μM 2'-F-CTP, 10 mM $Mg(OAc)_2$, and 10 nM MTdT-evo in 1× TdT buffer and incubated at 37 °C for 6 min. d. Incorporation of 2'-F-GTP. 100 nM primer FAM-18G was mixed with 0-64 μM 2'-F-GTP, 10 mM $Mg(OAc)_2$, and 4 nM MTdT-evo in 1× TdT buffer and incubated at 37 °C for 2 min. The products were analyzed with 20% denaturing PAGE gels supplemented with 8 M urea.

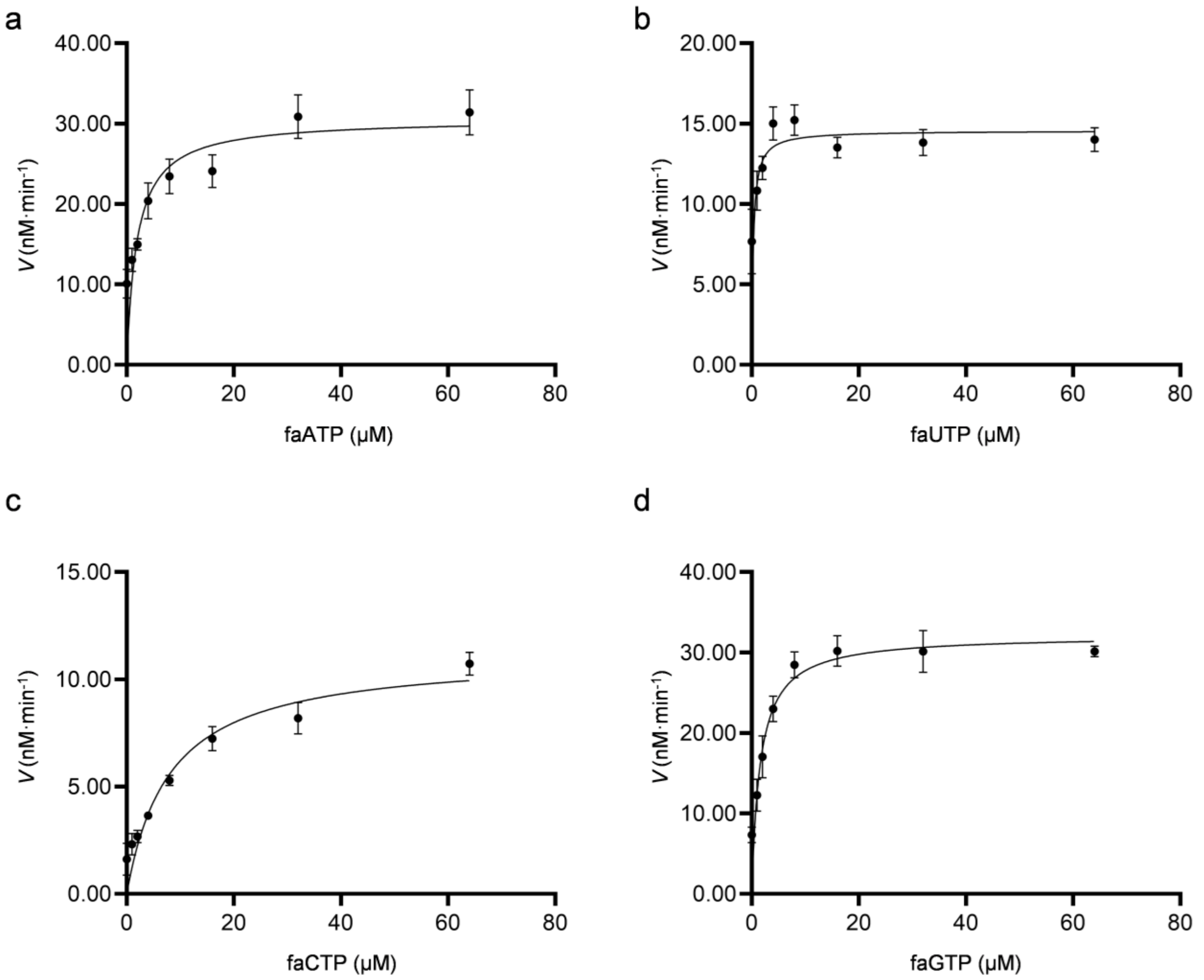

Figure S10. Michaelis-Menten plots for MTdT-evo-mediated incorporation of faNTPs. a. Incorporation of faATP. 100 nM primer FAM-18G was mixed with 0-64 μM faATP, 10 mM $Mg(OAc)_2$, and 2 nM MTdT-evo in 1× TdT buffer and incubated at 37 °C for 30 s. b. Incorporation of faUTP. 100 nM primer FAM-18G was mixed with 0-64 μM faUTP, 10 mM $Mg(OAc)_2$, and 2 nM MTdT-evo in 1× TdT buffer and incubated at 37 °C for 1 min. c. Incorporation of faCTP. 100 nM primer FAM-18G was mixed with 0-64 μM faCTP, 10 mM $Mg(OAc)_2$, and 1.5 nM MTdT-evo in 1× TdT buffer and incubated at 37 °C for 2 min. d. Incorporation of faGTP. 100 nM primer FAM-18G was mixed with 0-64 μM faGTP, 10 mM $Mg(OAc)_2$, and 2 nM MTdT-evo in 1× TdT buffer and incubated at 37 °C for 30 s. The products were analyzed with 20% denaturing PAGE gels supplemented with 8 M urea.

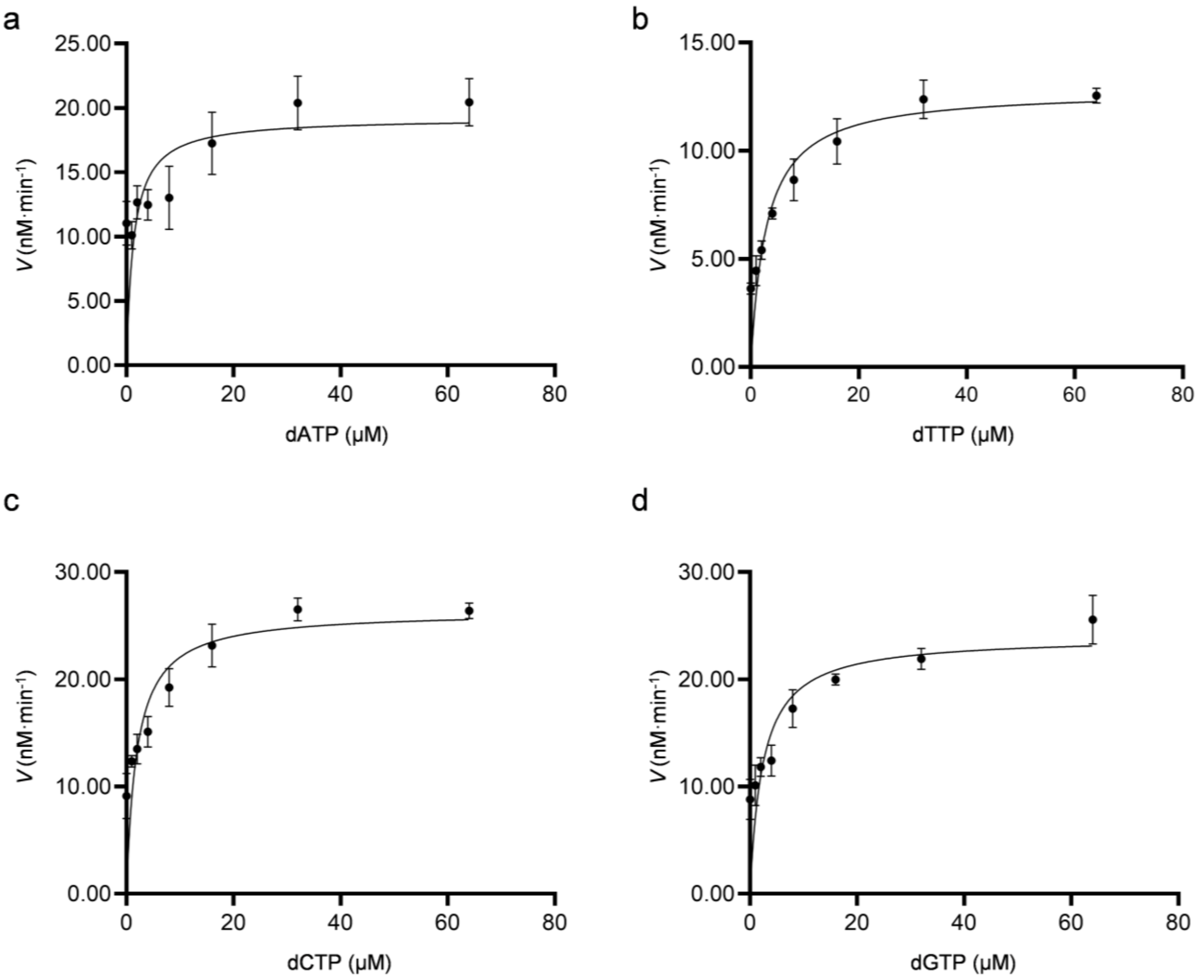

Figure S11. Michaelis-Menten plots for MTdT-evo-mediated incorporation of dNTPs. a. Incorporation of dATP. 100 nM primer FAM-18G was mixed with 0-64 μM dATP, 10 mM $Mg(OAc)_2$, and 3 nM MTdT-evo in 1× TdT buffer and incubated at 37 °C for 1 min. b. Incorporation of dTTP. 100 nM primer FAM-18G was mixed with 0-64 μM dTTP, 10 mM $Mg(OAc)_2$, and 3 nM MTdT-evo in 1× TdT buffer and incubated at 37 °C for 2 min. c. Incorporation of dCTP. 100 nM primer FAM-18G was mixed with 0-64 μM dCTP, 10 mM $Mg(OAc)_2$, and 3 nM MTdT-evo in 1× TdT buffer and incubated at 37 °C for 1 min. d. Incorporation of dGTP. 100 nM primer FAM-18G was mixed with 0-64 μM dGTP, 10 mM $Mg(OAc)_2$, and 3 nM MTdT-evo in 1× TdT buffer and incubated at 37 °C for 1 min. The products were analyzed with 20% denaturing PAGE gels supplemented with 8 M urea.

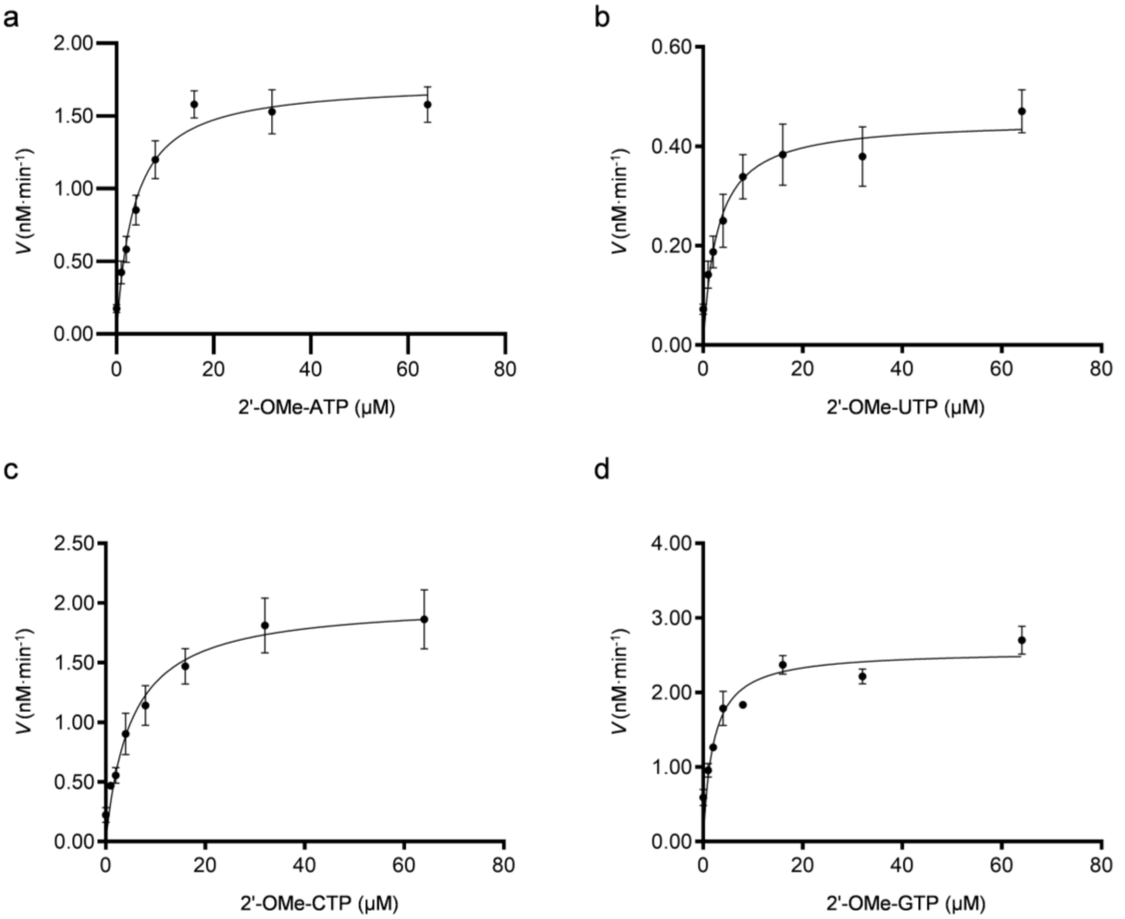

Figure S12. Michaelis-Menten plots for murine TdT-mediated incorporation of 2'-OMe-NTPs. a. Incorporation of 2'-OMe-ATP. 100 nM primer FAM-18G (Table S1) was mixed with 0-64 μM 2'-OMe-ATP, 10 mM $Mg(OAc)_2$, and 10 nM murine TdT in 1× TdT buffer and incubated at 37 °C for 20 min. b. Incorporation of 2'-OMe-UTP. 100 nM primer FAM-18G was mixed with 0-64 μM 2'-OMe-UTP, 10 mM $Mg(OAc)_2$, and 60 nM murine TdT in 1× TdT buffer and incubated at 37 °C for 62 min. c. Incorporation of 2'-OMe-CTP. 100 nM primer FAM-18G was mixed with 0-64 μM 2'-OMe-CTP, 10 mM $Mg(OAc)_2$, and 30 nM murine TdT in 1× TdT buffer and incubated at 37 °C for 20 min. d. Incorporation of 2'-OMe-GTP. 100 nM primer FAM-18G was mixed with 0-64 μM 2'-OMe-GTP, 10 mM $Mg(OAc)_2$, and 20 nM murine TdT in 1× TdT buffer and incubated at 37 °C for 10 min. The products were analyzed with 20% denaturing PAGE gels supplemented with 8 M urea.

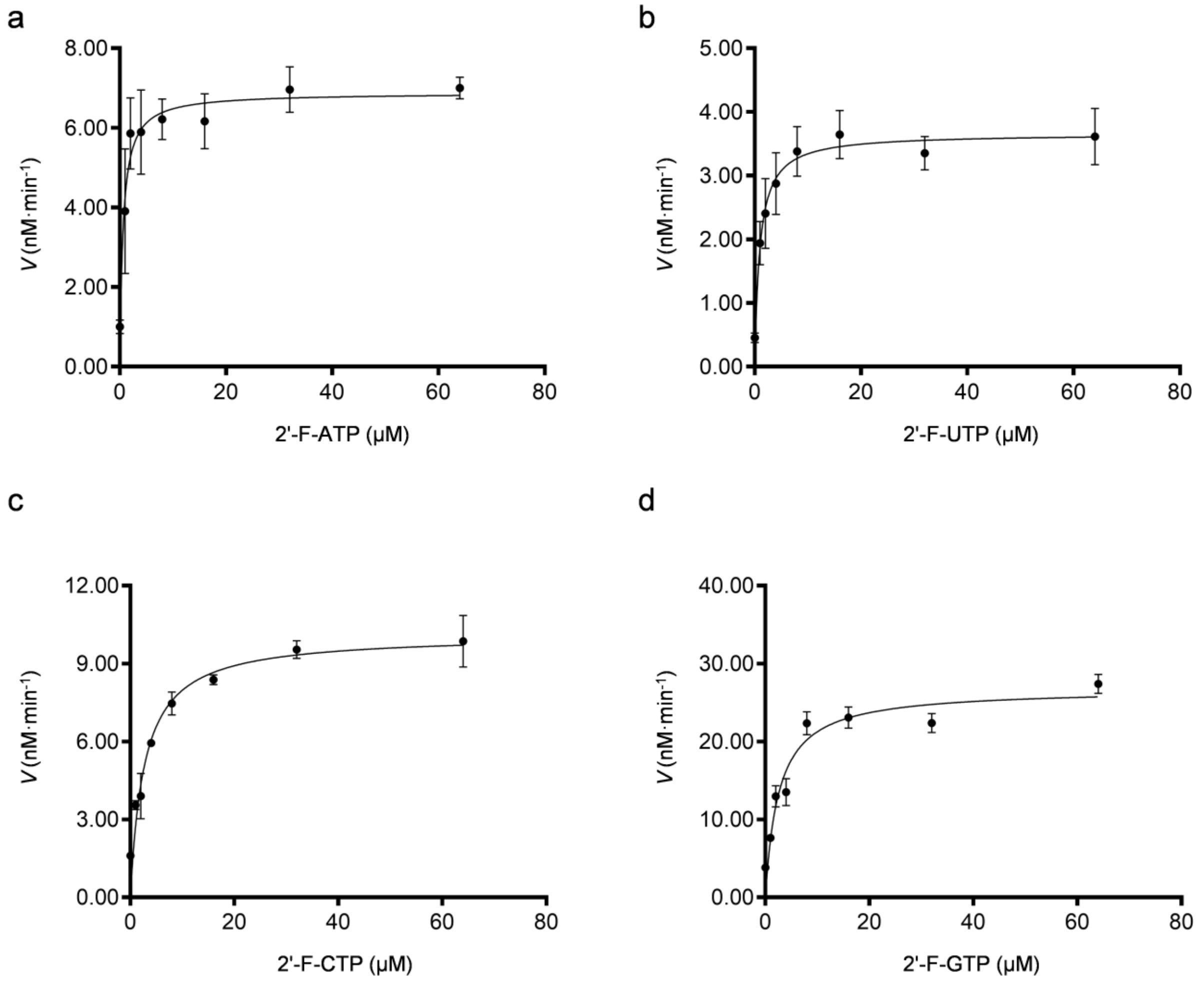

Figure S13. Michaelis-Menten plots for murine TdT-mediated incorporation of 2'-F-NTPs. a. Incorporation of 2'-F-ATP. 100 nM primer FAM-18G was mixed with 0-64 μM 2'-F-ATP, 10 mM $Mg(OAc)_2$, and 5 nM murine TdT in 1× TdT buffer and incubated at 37 °C for 5 min. b. Incorporation of 2'-F-UTP. 100 nM primer FAM-18G was mixed with 0-64 μM 2'-F-UTP, 10 mM $Mg(OAc)_2$, and 10 nM murine TdT in 1× TdT buffer and incubated at 37 °C for 10 min. c. Incorporation of 2'-F-CTP. 100 nM primer FAM-18G was mixed with 0-64 μM 2'-F-CTP, 10 mM $Mg(OAc)_2$, and 5 nM murine TdT in 1× TdT buffer and incubated at 37 °C for 3 min. d. Incorporation of 2'-F-GTP. 100 nM primer FAM-18G was mixed with 0-64 μM 2'-F-GTP, 10 mM $Mg(OAc)_2$, and 5 nM murine TdT in 1× TdT buffer and incubated at 37 °C for 1 min. The products were analyzed with 20% denaturing PAGE gels supplemented with 8 M urea.

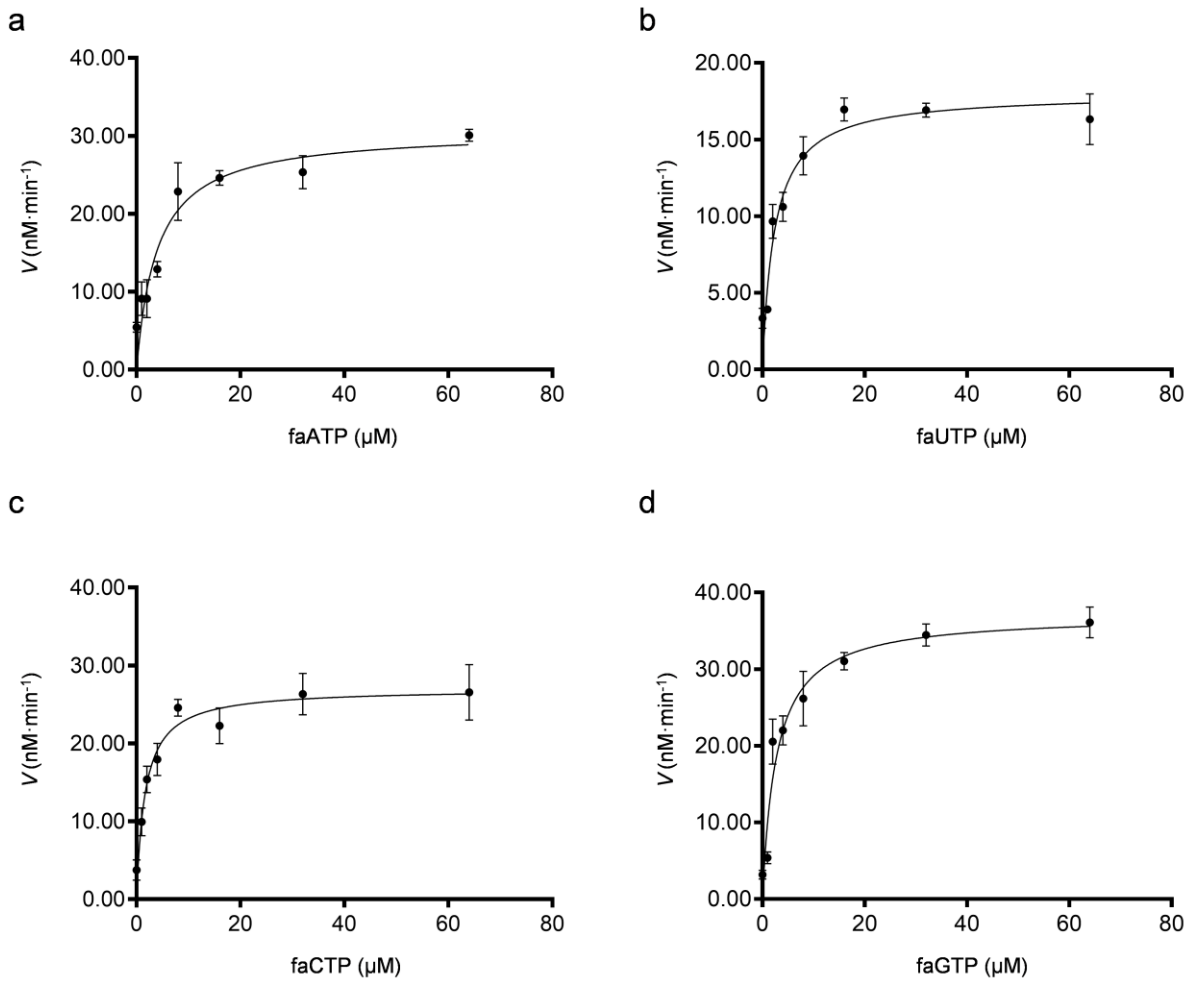

Figure S14. Michaelis-Menten plots for murine TdT-mediated incorporation of faNTPs. a. Incorporation of faATP. 100 nM primer FAM-18G was mixed with 0-64 μM faATP, 10 mM $Mg(OAc)_2$, and 2.5 nM murine TdT in 1× TdT buffer and incubated at 37 °C for 1 min. b. Incorporation of faUTP. 100 nM primer FAM-18G was mixed with 0-64 μM faUTP, 10 mM $Mg(OAc)_2$, and 5 nM murine TdT in 1× TdT buffer and incubated at 37 °C for 1.5 min. c. Incorporation of faCTP. 100 nM primer FAM-18G was mixed with 0-64 μM faCTP, 10 mM $Mg(OAc)_2$, and 5 nM murine TdT in 1× TdT buffer and incubated at 37 °C for 1 min. d. Incorporation of faGTP. 100 nM primer FAM-18G was mixed with 0-64 μM faGTP, 10 mM $Mg(OAc)_2$, and 2.5 nM murine TdT in 1× TdT buffer and incubated at 37 °C for 1 min. The products were analyzed with 20% denaturing PAGE gels supplemented with 8 M urea.

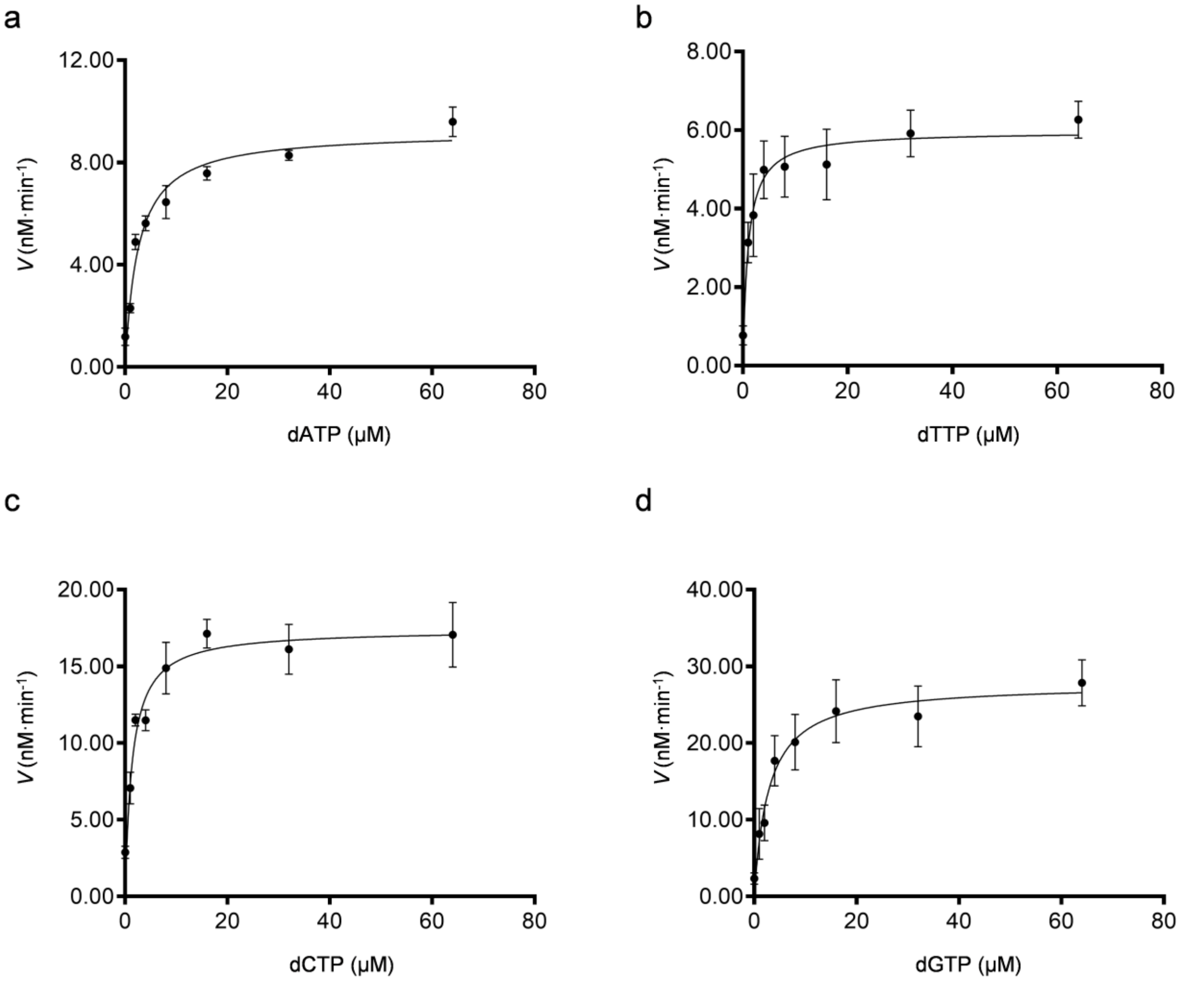

Figure S15. Michaelis-Menten plots for murine TdT-mediated incorporation of dNTPs. a. Incorporation of dATP. 100 nM primer FAM-18G was mixed with 0-64 µM dATP, 10 mM $Mg(OAc)_2$, and 5 nM murine TdT in 1× TdT buffer and incubated at 37 °C for 3 min. b. Incorporation of dTTP. 100 nM primer FAM-18G was mixed with 0-64 µM dTTP, 10 mM $Mg(OAc)_2$, and 5 nM murine TdT in 1× TdT buffer and incubated at 37 °C for 5 min. c. Incorporation of dCTP. 100 nM primer FAM-18G was mixed with 0-64 µM dCTP, 10 mM $Mg(OAc)_2$, and 5 nM murine TdT in 1× TdT buffer and incubated at 37 °C for 1.5 min. d. Incorporation of dGTP. 100 nM primer FAM-18G was mixed with 0-64 µM dGTP, 10 mM $Mg(OAc)_2$, and 5 nM murine TdT in 1× TdT buffer and incubated at 37 °C for 1 min. The products were analyzed with 20% denaturing PAGE gels supplemented with 8 M urea.

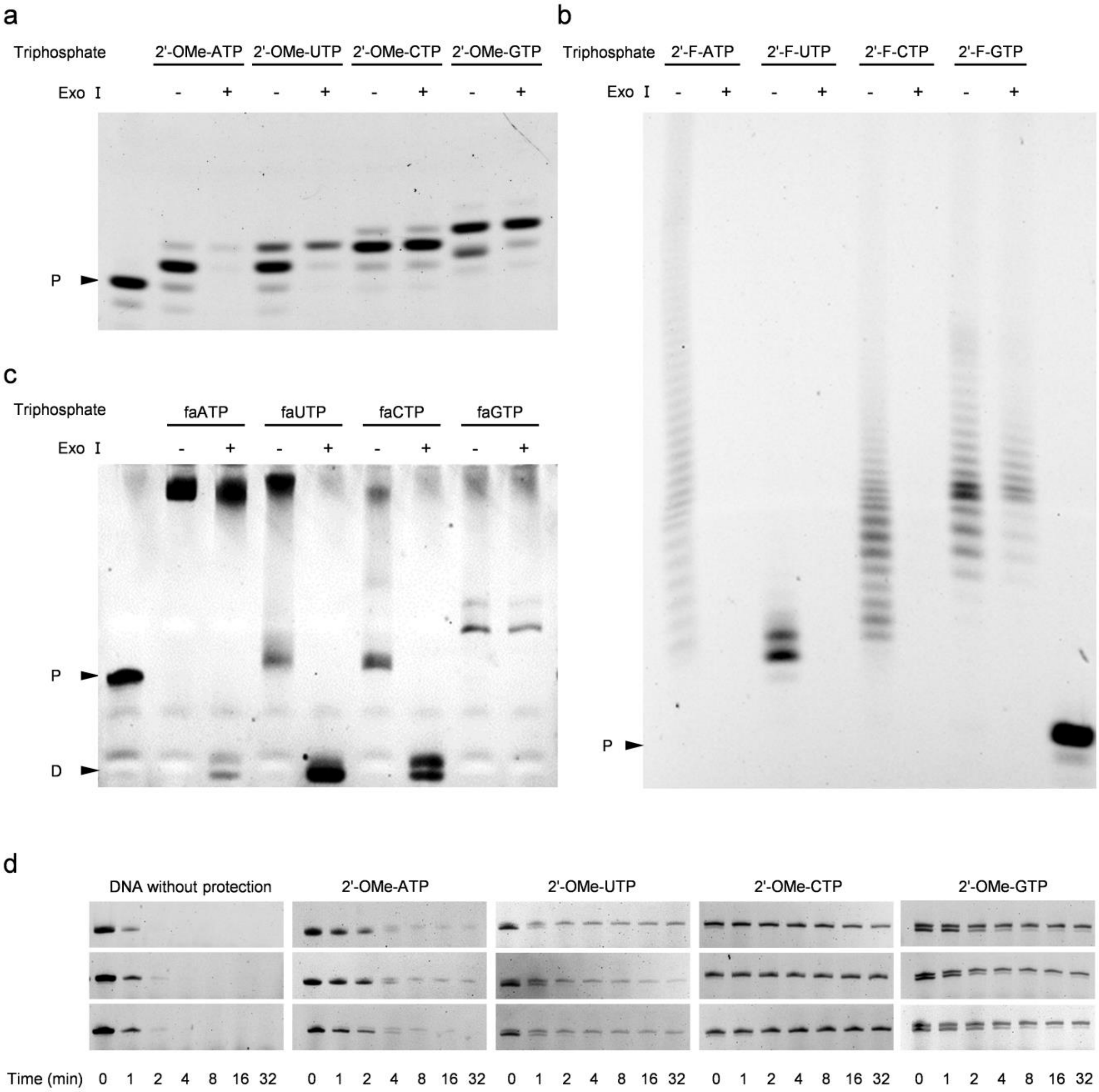

Figure S16. Resistances to exonuclease I (Exo I) of ssDNAs extended with different 2'-modified NTPs by MTdT-evo. a. ssDNAs extended with 2'-OMe-NTPs. b. ssDNAs extended with 2'-F-NTPs. c. ssDNAs extended with faNTPs. d. Time courses of Exo I degradation of ssDNA and ssDNAs extended with 2'-OMe-NTPs. For each reaction, 100 nM primer FAM-18G was mixed with 100 μM one of 2'-modified-NTPs, 1 mM $CoCl_2$,1 μM MTdT-evo in 1× TdT buffer and incubated at 37 °C for 60 min. After purification, the extension products were digested by incubation with 2 U/μL Exo I at 37°C for 15 min. The products were then analyzed with 20% denaturing PAGE gels supplemented with 8 M urea. P: primer FAM-18G. D: digestion product.

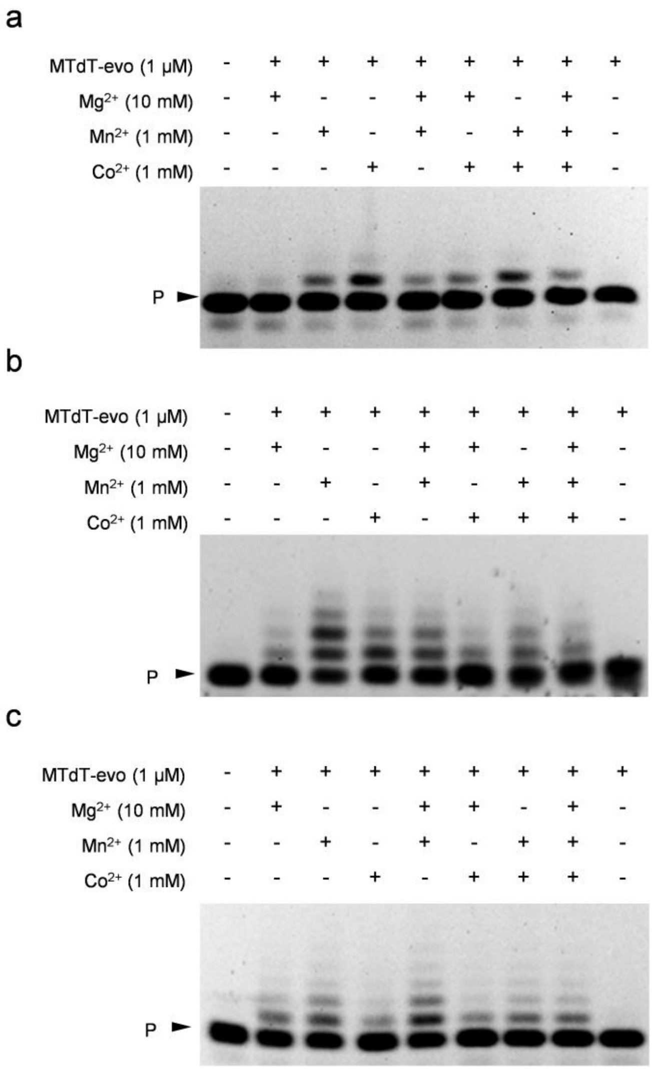

Figure S17. Effects of metal ions on MTdT-evo-mediated 3' tailing of dsDNA with different 2'-modified CTPs. a. 3' tailing with 2'-OMe-CTP. b. 3' tailing with 2'-F-CTP. c. 3' tailing with faCTP. For each reaction, 100 nM dsDNA was mixed with 100 μM one of 2'-modified CTPs and 1 μM MTdT-evo in 1× TdT buffer with 1 mM $CoCl_2$, 1 mM $MnCl_2$, 10 mM $Mg(OAc)_2$, or one of their combinations, and incubated at 37 °C for 60 min. The products were analyzed with 20% denaturing PAGE gels supplemented with 8 M urea. P: primer FAM-18G.

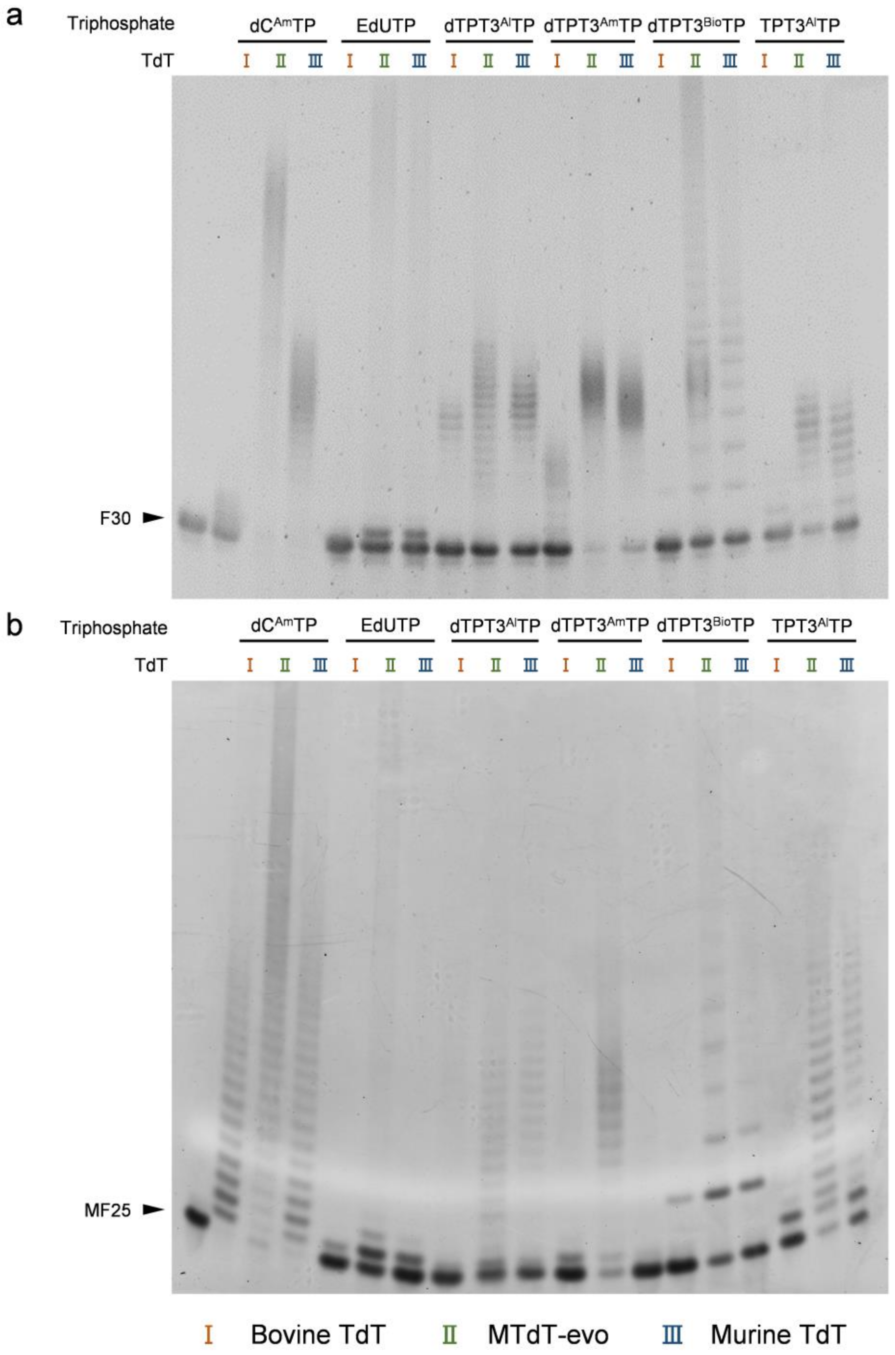

Figure S18. TdT-mediated extension of ssXNAs with (d)NTP derivatives containing a functionalized natural or unnatural nucleobase. a. Extension of single-stranded 2'-F-DNA F30 with functionalized (d)NTP derivatives. b. Extension of single-stranded 2'-OMe/F-DNA MF25 with functionalized (d)NTP derivatives. For each reaction, 100 nM ssXNA was mixed with 10 μM one of the functionalized (d)NTP derivatives, 1 mM $CoCl_2$, and 0.1 μM TdT in 1× TdT buffer and incubated at 37 °C for 30 min. The products were analyzed with 20% denaturing PAGE gels supplemented with 8 M urea.

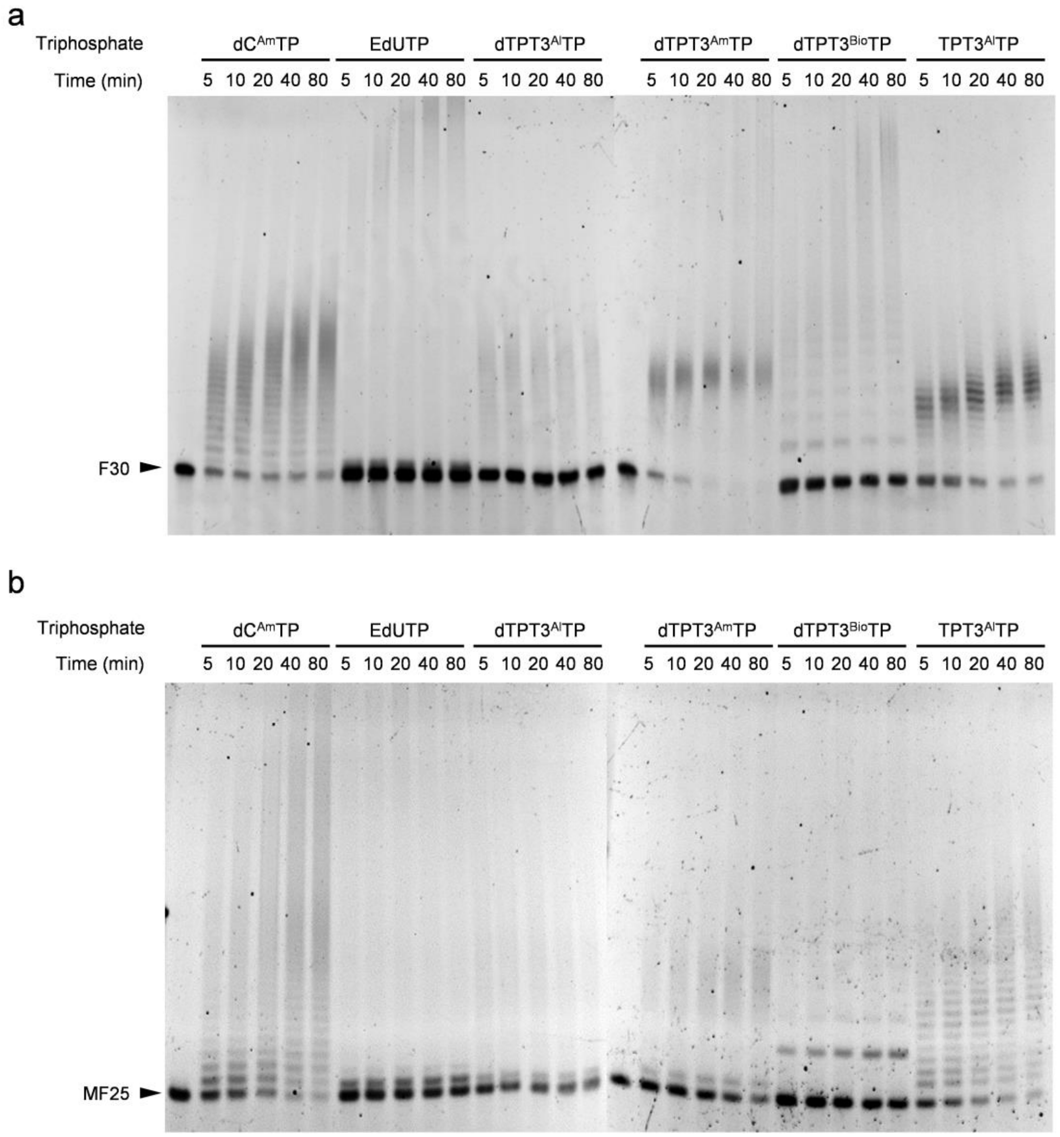

Figure S19. Time courses of MTdT-evo-mediated ssXNA extension with different functionalized nucleoside triphosphates. a. Extension of single-stranded 2'-F-DNA F30 with functionalized (d)NTP derivatives. b. Extension of single-stranded 2'-OMe/F-DNA MF25 with functionalized (d)NTP derivatives. For each reaction, 100 nM ssXNA was mixed with 10 μM one of the functionalized (d)NTP derivatives, 1 mM $CoCl_2$, and 0.1 μM MTdT-evo in 1× TdT buffer and incubated at 37 °C for 30 min. The products were analyzed with 20% denaturing PAGE gels supplemented with 8 M urea.

Supplementary Table

Table S1. Oligonucleotides used in this work.

Name	Sequence
FAM-18G	5'-FAM-ATACGACTCACTATAGGG
FAM-18A	5'-FAM-ATACGACTCACTATAAAA
FAM-18T	5'-FAM-ATACGACTCACTATATTT
FAM-18C	5'-FAM-ATACGACTCACTATACCC
18G-RC	5'-CCCTATAGTGAGTCGTAT
X_1	5'-CGACCGATGAATAGCGGTCAGATCCGTACCTACTCG
X_2	5'-CGAGTAGGTACGGATCTGCGTATTGCGAACGACTCG
X_3	5'-CGAGTCGTTCGCAATACGGCTGTACGTATGGTCTCG
X_4	5'-CGAGACCATACGTACAGCACCGCTATTCATCGGTCG
F30	5'-fCfAfGfTfAfGfTfCfTfAfGfCfAfCfTfTfTfCfAfGfCfTfTfTfG fTfTfCfCfC
MF25	5'-mGmGmGmAmAfCmAmAmAmGfCfUmGmAmAmGfUmA fCfUfUmAfCfCfC

*f: 2'-F-modified nucleotide; m: 2'-OMe-modified nucleotide; FAM: FAM-labelled.

Supplementary Note 1

Protein sequences

Bovine TdT (1)

MGSSHHHHHHGSGLVPRGSASMSDSEVNQEAKPEVKPEVKPETHINLKVSDGSSEIFF
KIKKTTPLRRLMEAFAKRQGKEMDSLRFLYDGIRIQADQTPEDLDMEDNDIIEAHREQ
IGGELMRTDYSATPNPGFQKTPPLAVKKISQYACQRKTTLNNYNHIFTDAFEILAENS
EFKENEVSYVTFMRAASVLKSLPFTIISMKDTEGIPCLGDKVKCIIEEIIEDGESSEVKA
VLNDERYQSFKLFTSVFGVGLKTSEKWFRMGFRSLSKIMSDKTLKFTKMQKAGFLY
YEDLVSCVTRAEAEAVGVLVKEAVWAFLPDAFVTMTGGFRRGKKIGHDVDFLITSP
GSAEDEEQLLPKVINLWEKKGLLLYYDLVESTFEKFKLPSRQVDTLDHFQKCFLILKL
HHQRVDSSKSNQQEGKTWKAIRVDLVMCPYENRAFALLGWTGSRQFERDIRRYATH
ERKMMLDNHALYDKTKRVFLKAESEEEIFAHLGLDYIEPWERNA

MTdT-evo (2)

MGHHHHHHHHHHSSGHIDDDDKHMMKIEEGKLVIWINGDKGYNGLAEVGKKFEKD
TGIKVTVEHPDKLEEKFPQVAATGDGPDIIFWAHDRFGGYAQSGLLAEITPDKAFQDK
LYPFTWDAVRYNGKLIAYPIAVEALSLIYNKDLLPNPPKTWEEIPALDKELKAKGKSA
LMFNLQEPYFTWPLIAADGGYAFKYENGKYDIKDVGVDNAGAKAGLTFLVDLIKNK
HMNADTDYSIAEAAFNKGETAMTINGPWAWSNIDTSKVNYGVTVLPTFKGQPSKPF
VGVLSAGINAASPNKELAKEFLENYLLTDEGLEAVNKDKPLGAVALKSYEEELVKDP
RIAATMENAQKGEIMPNIPQMSAFWYAVRTAVINAASGRQTVDEALKDAQTNSSSN
NNNNNNNNNLGIEGRISHMSMGGRDIVDGSEFSPSPVPGSQNVPAPAVKKISQYACQ
RRTTLNNYNQLFTDALEILAENAEFRENEGRCLAFMRAASVLKSLPFPITSMKDLEGL
PCLGDKVKRIIEEILEDGESSEAKAVLNDERYKSFKLFTSVFGVGLKTAEKWYRMGFR
TLSKIQSDKSLRLTQMQKAGFLYYEDLVSCVNRPEAEAVSMLVKEAVVTFLPGALVT
LTGGFRRGKMTGHDVDFLITSPEAGEDEEQQLLHKVTDFWKQQGLLLYCDILESTFE
KFKQPSRKVDALDHFQKCFLILKLDHGRVHSEKSGQQEGKGWKAIRVDLVMCPYDR
RAFALLGWTGSRQFERDLRRYATHERKMMLDNHALYDRTKRVFLEAESEEEIFAHL
GLDYIEPWERNA

Murine TdT (3)

MGSSHHHHHHSSGLVPRGSHMASMTGGQQMGRGSEFMDPLQAVHLGPRKKRPRQL

GTPVASTPYDIRFRDLVLFILEKKMGTTRRAFLMELARRKGFRVENELSDSVTHIVAE

NNSGSDVLEWLQLQNIKASSELELLDISWLIECMGAGKPVEMMGRHQLVVNRNSSPS

PVPGSQNVPAPAVKKISQYACQRRTTLNNYNQLFTDALDILAENDELRENEGSCLAF

MRASSVLKSLPFPITSMKDTEGIPCLGDKVKSIIEGIIEDGESSEAKAVLNDERYKSFKL

FTSVFGVGLKTAEKWFRMGFRTLSKIQSDKSLRFTQMQKAGFLYYEDLVSCVNRPEA

EAVSMLVKEAVVTFLPDALVTMTGGFRNGKMTGHDVDFLITSPEATEDEEQQLLHK

VTDFWKQQGLLLYCDILESTFEKFKQPSRKVDALDHFQKCFLILKLDHGRVHSEKSG

QQEGKGWKAIRVDLVMCPYDRRAFALLGWTGSAQFNRDLRRYATHERKMMLDNH

ALYDRTKRVFLEAESEEEIFAHLGLDYIEPWERNA

Supplementary Note 2

Expression and purification of terminal deoxynucleotidyl transferases (TdTs). TdTs used in this study were expressed and purified in the laboratory. A single colony of *Escherichia coli* (*E. coli*) BL21(DE3) cells harboring expression plasmids pET30a-Bovine TdT, pET28a-MTdT-evo, or pET28a-Murine TdT was inoculated in 2× YT medium (10 g/L yeast extract, 16 g/L tryptone and 5 g/L NaCl) supplemented with 50 µg/mL kanamycin and grown at 37 °C overnight. The overnight culture was re-inoculated at 1:100 dilution into fresh 2× YT medium supplemented with 50 µg/mL kanamycin and grown at 37 °C. When OD_{600} of the culture reached 0.6-0.8, 0.4 mM IPTG was added to induce protein expression, and the culture was then grown at 16 °C for 20 h. The cells were harvested by centrifugation at 4,500 rpm and 4 °C, resuspended in 1× binding buffer (50 mM Tris-HCl, 5 mM imidazole, 0.1 mM EDTA, 150 mM NaCl, 0.1% Triton X-100, pH 7.5), and disrupted with a high-pressure homogenizer. The cell lysate was centrifuged at 10,000 rpm and 4 °C for 60 min, and the supernatant was then filtrated successively with 0.45 µm and 0.22 µm membrane filters. The filtered supernatant was loaded onto a Ni-NTA sefinose resin 6FF column. The column was washed with 10 column volumes of washing buffer (50 mM Tris-HCl, 10 mM imidazole, 0.1 mM EDTA, 300 mM NaCl, 0.1% Triton X-100, pH 7.5) and the protein was then eluted with elution buffer (50 mM Tris-HCl, 50-500 mM imidazole, 0.1 mM EDTA, 150 mM NaCl, 0.1% Triton X-100, 1 mM DTT, pH 7.5). The purified protein (in elution fractions of 150 mM and 300 mM imidazole) was then concentrated and dialyzed with an Amicon Ultra-15 centrifugal filter (50 kDa). The purified and concentrated bovine TdT, MTdT-evo and murine TdT proteins were quantified with a BCA protein assay kit according to the manufacturer's guidance.

References

1 Chua, J.P.S., Go, M.K., Osothprarop, T., Mcdonald, S., Karabadzhak, A.G., Yew, W.S., Peisajovich, S. and Nirantar, S. (2020) Evolving a thermostable terminal deoxynucleotidyl transferase. *ACS Synth. Biol.*, **9**, 1725-1735.

2 Barthel, S., Palluk, S., Hillson, N.J., Keasling, J.D. and Arlow, D.H. (2020) Enhancing terminal deoxynucleotidyl transferase activity on substrates with 3' terminal structures for enzymatic *de novo* DNA synthesis. *Genes-Basel*, **11**, 102.

3 Ybert, T. and Delarue, M. (2017). New variant of DNA polymerase of polX family comprising mutation of amino acid residue at specific position, useful for synthesizing template-free nucleic acid molecule and strand of DNA or RNA. FR3052462-A1. (Patent).
